# Hope, Optimism, and Expectations for the Political Future

**DOI:** 10.1007/s11109-025-10027-5

**Published:** 2025-03-13

**Authors:** Matthew Barnfield, Rob Johns

**Affiliations:** 1 School of Politics and International Relations, Queen Mary University of London, London, UK; 2 Department of Politics and International Relations, University of Southampton, Southampton, UK

**Keywords:** Optimism, Hope, Electoral expectations, Prospective evaluations, Wishful thinking

## Abstract

Expectations of what the future holds are a significant driver of political behavior. It is therefore important to understand the sources of those expectations. In this paper, we explore the psychological dispositions driving positivity about the political future. We draw on psychologists’ distinction between optimism—a dispositional belief that good things will happen, come what may—and hope—a trait of envisioning, pursuing and believing one’s goals to be achievable. We assess pre-registered hypotheses about the distinct influences of optimism and hope on valence expectations—beliefs about whether there will be good or bad societal outcomes—and electoral expectations—beliefs about likely election outcomes—in a representative-sample survey experiment in the United Kingdom. We find that optimism drives positive valence expectations, but hope drives partisan electoral expectations. Indeed, partisan bias in electoral expectations is exhibited only by those scoring higher in hope. We show experimentally that positive information from polls and expert commentary dampens this impact of hope by raising the expectations of the otherwise unhopeful. Our findings suggest that so-called ‘wishful thinking’ about election outcomes might stem from a sense of agency around electoral politics, but that this sense of agency does not extend to how citizens envision society’s prospects.

**Supplementary Information:**

The online version contains supplementary material available at 10.1007/s11109-025-10027-5.

## Introduction

To understand electoral politics, it is important to understand people’s expectations for the future. For one thing, democracy itself is structurally future-orientated, relying on the pursuit of uncertain goals and the possibility of shaping a better collective future (Calhoun et al. 2022; MacKenzie 2021; White 2024). For another, expectations also have concrete effects on voters’ assessments and strategies at election time. Most obviously, what we call *valence expectations*—that is, expectations of whether things will get better or worse in terms of shared societal goals like economic prosperity—matter electorally. For example, prospective evaluations of whether economic performance will improve or worsen are a powerful influence on vote choice (Elis, Hillygus, and Nie 2010; Lacy and Christenson 2017). *Electoral expectations*—predictions of likely election outcomes—matter too. Voters’ estimations of the likely results of an election shape their strategic and purposive choices at the ballot box (Blais et al. 2006), and partisans who incorrectly expect their side to win are sometimes liable to have especially negative reactions to electoral defeat (Krizan et al. 2010; Mongrain 2023), potentially compromising the ‘losers’ consent’ on which representative democracy depends (Anderson et al. 2005). The effects of valence expectations and electoral expectations may even interact: a voter’s assessment of how the economy will fare in the coming years is more likely to predict their vote choice if they correctly foresee who will win the election and be responsible for such economic change (Lacy and Christenson 2017). As Irwin and Van Holsteyn (2002, 92) conclude, ‘if expectations are central to vote decisions, it becomes as important to investigate the source of these expectations as to study the vote decision itself’.

One such source has long been very clear. There is a hefty component of partisan bias in prospective economic evaluations—a commonly measured form of valence expectation—with supporters of the incumbent expecting a more positive economic future (Anderson et al. 2004; Conover, Feldman, and Knight 1987; Ladner and Wlezien 2007). Partisan bias is just as clearly on show when it comes to electoral expectations. The tendency for citizens to expect their preferred parties to win was remarked on by S. P. Hayes (1932) almost a century ago and remains a perennial finding (e.g. Babad 1995; Mongrain 2021; Searles et al. 2018; Tikochinski and Babad 2023). However, while party preference colors expectations, it does not fully determine them. Regardless of their preferences, there is variance in how likely people think it is that good outcomes will obtain in the future. This imperfect correlation raises a question: are there individual differences in dispositions to think positively about the political future?

We address that question in this paper and show that political expectations partly reflect the psychological dispositions of *optimism* and *hope*. Psychologists define optimism and hope as distinct psychological constructs (Alarcon et al. 2013; Bailey et al. 2007; Rand 2017). Optimism is a general and unconditional positivity in expectations about the future—things will work out in the end, come what may. By contrast, hope is for psychologists a trait of tending to believe that one’s goals can be achieved—when there’s a will, there’s a way.

We conduct a survey experiment on a nationally representative UK sample recruited via Prolific (N = 1,697). We find that optimism promotes more positivity in valence expectations but not electoral expectations. Hope, meanwhile, does not translate into upbeatness about wider national outcomes but it does boost expectations of one’s own party’s electoral prospects. Strikingly, there is no evidence of so-called ‘wishful thinking’ about election outcomes among those scoring low in hope: only the electoral expectations of the hopeful particularly favor their preferred party. In the experimental component of our study, exposing respondents to various cues intended to shift their electoral expectations, we again primarily find evidence of the influence of hope. Although in one case, optimism appears to have a slight positive moderating effect, this effect is not robust and is dwarfed by that of hope. This effect of hope, though, comes as a *negative* interaction effect whereby hopeful supporters—who already have higher expectations—respond less to upbeat forecasts for their preferred party than do the less hopeful supporters of the same party.

These findings reveal the different mechanisms driving valence and electoral expectations and, in doing so, may shed light on where in politics people feel a sense of agency. That the optimistic are positive about society’s prospects, while the hopeful are positive about their preferred election outcomes, suggests that people generally see the former as beyond their control, but the latter as a collective endeavor in which they partake. Our results also reveal that electoral preferences alone are not enough to drive the wishful thinking effect long observed in electoral expectations: supporters of a party need either such an internal, hopeful sense of agency, or some objective information from outside, to be convinced of likely victory.

### Optimism, Hope, and Political Expectations

In everyday language, optimism and hope are near synonyms, both denoting a positive attitude towards the future. However, psychologists emphasize a distinction between the two (Alarcon et al. 2013; Bailey et al. 2007; Carver and Scheier 2014). In personality psychology, optimism refers simply to the degree to which people generally believe that positive outcomes will predominate over negative outcomes (Scheier and Carver 1985). Alarcon et al. (2013, 822) explain that ‘the optimistic person believes that somehow… his or her future will be successful and fulfilling’. Optimism is agnostic as to *how* these positive outcomes will come about; it simply describes a generalised tendency to believe that they will come about, come what may.^1^

Psychologists define hope as a person’s tendency to believe that their goals can be achieved (Snyder 2002). Alarcon et al. (2013, 822) explain that ‘the hopeful person… believes specifically in his or her own capability for securing a successful and fulfilling future’. This definition is typically decomposed into two parts: *pathways* describe people’s ability to imagine many ways a good outcome could come about, and *agency* denotes the motivation to pursue those pathways (Babyak et al. 1993). Hopeful people see pathways to the things they want, and are willing to pursue them—and *because* of that, they believe their goals can be achieved (Pettit 2004). The notion of agency is therefore at the heart of hope (Cohen-Chen and Van Zomeren 2018), creating a virtuous circle: I pursue this outcome partly because I believe it is possible and I believe it is possible partly because I am pursuing it (Goldman 2012; 2023).

Now, there is a danger of overdrawing the difference between optimism and hope. They overlap both conceptually and in how they manifest empirically—as positive attitudes towards the future. Predictably then, the two are typically quite strongly correlated when measured in the same study. However, that correlation is far from perfect (e.g. Bailey et al. 2007). It is possible to be a hopeful pessimist, or a hopeless optimist. Moreover, the theoretical distinction between the two may link them rather differently to the two types of political expectation distinguished at the outset.

### Valence Expectations

When it comes to valence judgements, intuitively, we would expect that people who are more optimistic in general would hold correspondingly more upbeat beliefs about the political future (Stapleton, Oliver, and Wolak 2021). This intuition remains untested because studies and survey measures of optimism have typically focused on personal outcomes. However, since there is no obvious conceptual limit on optimism’s remit, we should expect it to apply to anything valenced—anything that can turn out good or bad in some easily identifiable sense. It is therefore reasonable to hypothesise^2^:

#### H_1OPT_:

There is a positive effect of optimism on politically relevant valence expectations.

The case for a relationship between hope and positive valence expectations is less clear-cut. Since hope is more self-directed in its emphasis on the individual’s agency in pursuing and achieving goals through various means (Alarcon et al. 2013), it should have more influence on expectations of outcomes over which people have more control and which are of particular importance to them, conceived as goals to be achieved. It would seem coherent for an individual scoring high in the psychological trait of hope nonetheless to see the political future as bleak, being out of their hands. Nonetheless, there is evidence that people do discern their own small contribution to societal outcomes. For example, recycling behaviour is driven quite powerfully by a sense of efficacy (Lauren et al. 2016; Tabernero and Hernández 2011), not because people imagine that their input will be pivotal but because they have a sense of contributing to a collective outcome. While the equivalent contribution to, say, economic growth may be rather less obvious or tangible, a similar mechanism may well apply. To account for this possibility, we also hypothesise:

#### H_1HOPE:_

There is a positive effect of hope on politically relevant valence expectations.

### Electoral Expectations

Meanwhile, research on electoral expectations—how well people think parties will perform at upcoming elections—has long shown that supporters of a party are much more likely to expect it to win (Mongrain 2021). This well-established empirical regularity known as ‘wishful thinking’ suggests that people have a tendency to expect positive electoral outcomes (Tikochinski and Babad 2023).

Given that optimism is itself the norm (Baranski et al. 2021), it is possible that this corresponding norm of expecting positive election outcomes is a result of generally optimistic dispositions. Certainly, scholars have often mentioned ‘optimism’ when discussing partisan electoral expectations (Babad et al. 1992; Babad 1997; Granberg and Holmberg 1988; Krizan and Sweeny 2013; Tenenboim-Weinblatt et al. 2022). It is therefore reasonable to conjecture that optimism drives electoral wishful thinking—or, more precisely, that it moderates the association between partisan preferences and electoral expectations:

#### H_2OPT_:

There is a positive interaction effect of optimism and partisan preferences on electoral expectations.

However, there are also good theoretical reasons to believe that hope would underpin positivity in partisan expectations. First, partisans play a role—however small—in achieving the goal of electing their party. They vote for their party or for a tactically savvy alternative, they might persuade others to do so, or they might make campaign donations. More hopeful people should be more inclined to believe that these acts play a role in boosting their party’s chances of winning, if hope is about seeing and pursuing pathways to achieve goals.

Second, and related, partisanship forms a stable part of many people’s identities (Green et al. 2002; Huddy et al. 2015) such that they are likely to see partisan election outcomes essentially as personal goals. In other words, hopeful partisans identify as part of something bigger, and may therefore apply their hopeful tendencies for their own futures to the future of the party as a result. This account sounds particularly applicable to those who identify quite strongly with a party—indeed, one item in a common measure of partisanship as a social identity asks “When talking about Democrats/Republicans, how often do you say ‘we’ instead of ‘they’?” (Huddy et al. 2015). However, the literature on wishful thinking suggests that the ‘partisan electoral expectations’ referred to in this paper may not be confined only to those with the strongest and most longstanding loyalties to parties. Studies show that simply preferring a particular party to win, or intending to vote for that party, is sufficient to skew expectations in its favor (Meffert et al. 2011; Searles et al. 2018; Leiter et al. 2018). The ‘partisan’ in ‘partisan electoral expectations’ can thus be more broadly interpreted as having taken a side in an electoral contest.

Finally, hope partly captures individuals’ ability to see different routes to good future outcomes, and one such route might be the election of their preferred party. If people are hopeful about those outcomes, then they must (by psychologists’ definition of hope) see ways to achieve them, and it would make sense for them to consider their preferred party and its policies as one such pathway. For all these reasons, we specify a parallel hypothesis in the case of hope:

#### H_2HOPE_:

There is a positive interaction effect of hope and partisan preferences on electoral expectations.

These hypotheses concern the baseline effects of the two psychological predispositions on electoral expectations. A further possibility is that optimism and hope also shape the way that people respond to incoming information relevant to the likely election outcome. Although expectations are deeply colored by electoral preferences, consistent evidence has demonstrated that citizens are at least somewhat responsive to such information. For example, people who pay more attention to politics tend to have expectations more consistent with the polling picture (Irwin and Van Holsteyn 2002; Meffert et al. 2011), voters report getting most of their information from polls (Lavrakas, Holley, and Miller 1991), and expectations even appear to respond to small changes between polls over time (Barnfield 2023a). Experimental evidence also shows that people make use of election forecasts (Barnfield et al. Forthcoming; Westwood et al. 2020) and commentators’ projections (Searles et al. 2018), when available. Although it is possible that responses to informational cues may vary depending on whether they require updating expectations for their preferred (in-)party or their less-preferred (out-)party (Tikochinski and Babad 2023), it is nonetheless likely that all such signals affect expectations in the relevant direction:

#### H_3_:

In-party-boosting signals have a positive effect on electoral expectations for the in-party.

#### H_4_:

Out-party-boosting signals have a negative effect on electoral expectations for the in-party.

These main effects of information are not our primary concern here, however. Our question is whether the psychological predispositions of optimism and hope *bias* the interpretation of this information. For example, optimistic partisans may be more responsive than pessimistic partisans to cues indicating that their party has a chance of winning the election. Hopeful partisans may be less responsive than their less hopeful counterparts to information indicating that the opposing party is in pole position. If optimism is a tendency to expect things to turn out well, and hope is a tendency to see ways to achieve goals, we might expect the dispositionally optimistic and the hopeful to be more attentive to good signs than bad (see Tiberius 2008). On this reading, optimism and hope would amplify the effect of positive signals for the in-party and dampen the effect of positive signals for the out-party—translating, in both cases, to positive interaction effects between the treatment effects in H_3_/H_4_ and the relevant psychological variable:

#### H_3OPT/HOPE_:

The interaction effect of in-party-boosting signals and optimism/hope on electoral expectations is positive.

#### H_4OPT/HOPE_:

The interaction effect of out-party-boosting signals and optimism/hope on electoral expectations is positive.

## Data and Methods

### Sample

To assess these pre-registered hypotheses, we used an online survey with an embedded experiment. The survey was fielded online between 13 and 17 March 2023 on a demographically representative sample of British adults (N = 1,697) recruited by the panel provider Prolific. This agency is now commonly used for research in political psychology (Bizumic et al. 2021; Greenaway 2023), delivering either convenience samples or, as in this case, a non-probability quota-based sample census-matched on age ($M=$ 45.85, $\sigma =$ 15.7), sex (48.6% men, 51.4% women), and ethnicity (87.3% white, 7.3% Asian, 3.12% black, 1.4% mixed, 0.9% other).^3^ The study received ethical approval from the University of Essex Ethics Sub Committee 3 (ETH2223-0304). We paid participants £1.20 for completing a survey of an estimated 8 min in length.^4^ They provided informed consent to participate in the study and have the results published in aggregated form.

### Measures

Our survey included measures of dispositional optimism, agency and pathway hope, political preferences, valence expectations, and electoral expectations.

#### Optimism and Hope

Dispositional optimism was measured using the Life Orientation Test-Revised (LOT-R) (Scheier et al. 1994). We used the standard five-point response scale from strongly disagree to agree (Baranski et al. 2021). Some items are reverse coded—e.g., ‘I hardly ever expect things to go my way’. The full scale, which also included the recommended filler items, is reported in the Supplementary Material. In our analyses, we use each respondent’s normalised mean response ($M=$ 0.56, $\sigma =$ 0.22) as their measure of optimism.

To measure hope, we used the Adult Hope Scale (Babyak et al. 1993), including both the agency (e.g. ‘My past experiences have prepared me well for my future’) and pathway (e.g. ‘There are lots of ways around any problem’) subscales. Again, we take each respondent’s normalised mean response across the whole scale ($M=$ 0.65, $\sigma =$ 0.17).^5^

Although optimism and hope are strongly correlated (*r* = 0.63), in the Supplementary Material we present the results of confirmatory factor analyses (CFA) showing that a model treating hope and optimism as separate constructs fits the data significantly better than one assuming the scales measure the same construct. These CFA results also show that decomposing the optimism scale into positively and negatively coded item subscales (albeit highly correlated subscales, *r* = −0.72), and decomposing the hope scale into agency and pathway subscales (again highly correlated at *r* = 0.72) results in even better overall fit. However, using one single optimism scale and one single hope scale also shows an acceptable fit to the data. For the sake of parsimony, we therefore present analyses using the full scales in our main results below, but we also present analyses with decomposed scales in the Supplementary Material. In all cases, results are consistent—i.e., conclusions we draw about the effect of hope based on analyses with the full scale below hold separately for both agency and pathway hope when the scale is decomposed. Finally, CFA also showed that an additional item we added to the optimism scale—‘this year will be better than last year for me’—substantially reduces fit, so we do not include it in our analysis.

#### Electoral Preferences

Following the example of Searles et al. (2018), our main measure of preference was a forced-choice question about only the Labour and Conservative parties, asking respondents ‘if you had to choose, which of the following political parties would you want to win the next general election?’ It is important to emphasize two related points about this measure. First, it is intentionally a measure of current electoral preference, not longstanding partisan identity. Second, as a result, the options were confined to the only two plausible winners of that upcoming election. The reasoning is straightforward: whether or not they identify with any party or none, most British citizens know that elections are highly likely to be won (whether with a majority or as the largest party) by Labour or the Conservatives, most have a preference among those two outcomes, and there is at least the potential for those preferences to shape electoral expectations. As noted earlier, studies on wishful thinking have suggested that preferences are sufficient to drive expectations—it does not necessarily require deep-rooted identities. But such bias is likely to be most powerful among the strongest partisans, and so we should acknowledge that our choice of a measure of preferences—including among those with no particular affinity for Labour or the Conservatives—may represent a more modest average effect than we would have observed had we confined the sample to the ‘most likely’ cases of Labour and Conservative party identifiers.

#### Valence Expectations

To test H_1_, our primary dependent variables measuring valence expectations take the form of a series of 0–10 ratings of how likely various outcomes are to happen ‘in the next few years’, from ‘very unlikely’ to ‘very likely’. By measuring these items on a very different outcome scale, we help to deter respondents from treating these questions simply as an extension of the optimism and hope scales they had seen previously. While the ratings were presented in random order, they were designed to form a balanced scale of four pairs, each comprising one desirable and one undesirable outcome:

- **Global evaluations**: *Positive*: the war in Ukraine will reach a peaceful resolution. *Negative*: there will be another pandemic.
- **National evaluations with implied government responsibility**: *Positive*: rates of poverty will decrease in the UK. *Negative*: the UK will undergo another recession.
- **National evaluations without implied government responsibility**: *Positive*: England’s football team will win the 2024 Euros. *Negative*: Team GB will fail to win any gold medals at the 2024 Olympics.
- **Personal evaluations**: *Positive*: you or someone in your family will get a big promotion at work. *Negative*: you or someone in your family will experience the end of a marriage or close relationship.

We measured five further valence expectations on a five-point ordinal scale (‘will get a lot worse’ to ‘will get a lot better’) mirroring the standard measurement of prospective economic evaluations. We ask respondents ‘how do you think each of these things will change over the coming year, before the next general election takes place?’ and then ‘how do you think each of these things will change over the years immediately after the next general election?’: the economic situation in the UK, the state of the NHS, the UK’s rate of carbon emissions, the quality of life in the UK, the global climate crisis. These items correspond more closely to the typical measurement of prospective economic evaluations in, e.g., the British Election Study.

#### Electoral Expectations

For testing H_2_, our primary measure of electoral expectations mirrors the standard item used in the British Election Study: ‘how likely do you think it is that each of the following parties will win more than half of the seats in the general election so it will be able to form a government on its own?’. Labour, the Conservatives and the Liberal Democrats were rated on a 0–10 scale ranging from ‘very unlikely’ to ‘very likely’. We use only Labour and Conservative ratings in our results below but discuss Liberal Democrat expectations in the Supplementary Material. In some of our analyses, our dependent variable is the advantage/disadvantage assigned to the Labour Party over the Conservative Party, taken by subtracting the perceived likelihood of a Conservative majority from the perceived likelihood of a Labour majority. Our second measure of electoral expectations is ‘which of the following outcomes do you think is most likely at the next general election?’: a Labour majority government, Conservative majority government, Labour-led coalition, or Conservative-led coalition. We analyze this outcome in the Supplementary Material to assess the robustness of our results. The Supplementary Material also explores two items asking respondents whether they think the Labour and Conservative Party will perform ‘much worse’, ‘somewhat worse’, ‘about the same’, ‘somewhat better’, or ‘much better’ than polls and experts say they will at the next general election.

### Experimental Component

We test H_3_ and H_4_ experimentally. Before asking participants to report their electoral expectations, we randomly split our sample into four groups. *Control* group ($N=$ 425) respondents were simply told ‘Now, we would like to know what you expect the result of the next general election will be’. This condition forms a baseline against which we assess the effects of our treatments.

The three treatment groups were presented with the results of a recent poll in which the Labour Party held a large lead over the Conservatives (YouGov 2023). We used real, up-to-date polling and commentary following recent work on the ethical and inferential advantages of ‘true treatments’ (Barnfield 2023b). The first of these treatment groups, the *Poll* condition ($N=$ 423), were simply shown that poll, allowing us to assess the main effect of exposure to a signal with clear implications for the outcome, and how this effect varied across levels of optimism and hope.

For the next group, *Poll w/ expert reinforcement* ($N=$ 424), the poll was accompanied by a reinforcing statement from a political expert: a quotation from Professor Will Jennings’s X/Twitter thread claiming that the Conservatives’ poor performance in the polls was unlikely to improve considerably before the election (Jennings 2022). The final, *Poll w/ expert doubt* group ($N=$ 425), saw the same poll but with an undermining statement, quoting an X/Twitter thread from Professor Rob Ford, another political expert, arguing that the Conservatives were likely to regain ground in the polls before the next general election (Ford 2022).^6^ These latter two conditions were included to assess whether expert reinforcement or questioning of a poll moderated its main effect on expectations and how far any moderating effect varied by levels of optimism and hope.

**Fig. 1:**
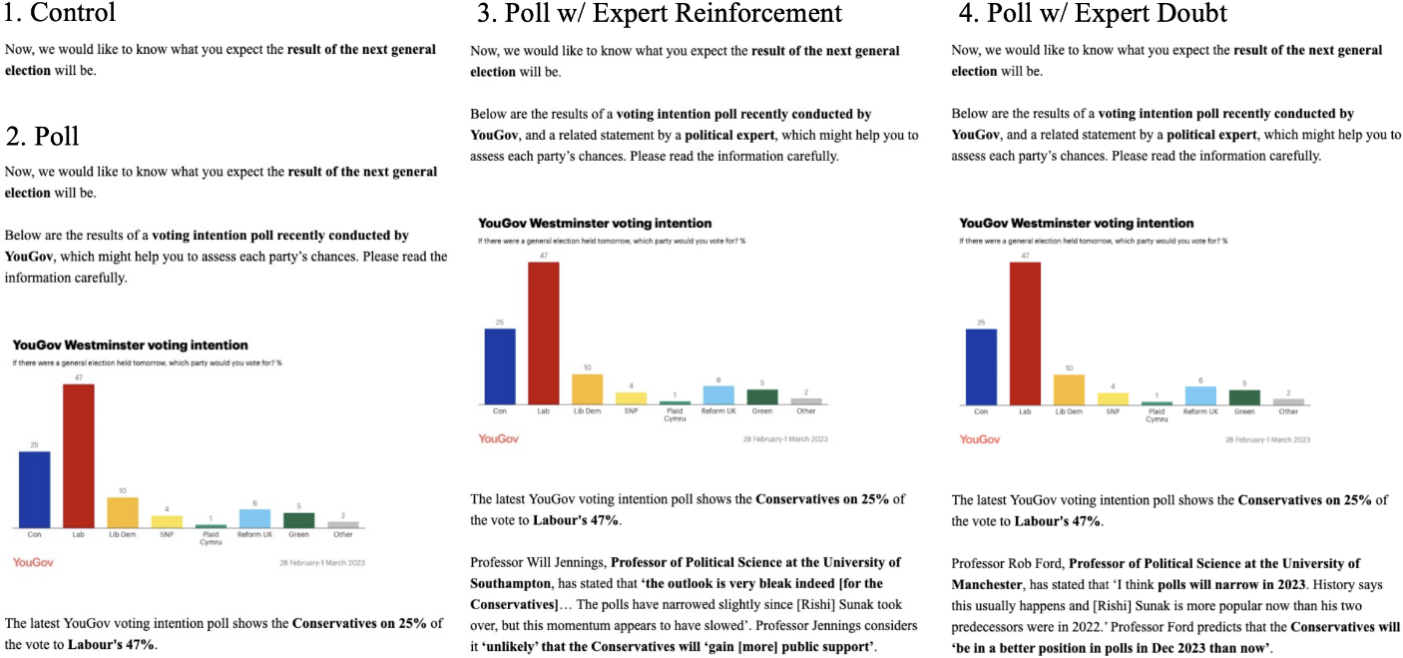
Randomly assigned treatment conditions in survey experiment

The screens displayed in these four conditions are shown in Fig. 1. Respondents in any of the three treatment conditions had to remain for 10 s on the screen displaying the information, in a bid to maximise their attentiveness.

### Analytical Approach

We assess our hypotheses primarily through a series of OLS regression models. We focus on presenting results visually below, and only report full regression tables in the Supplementary Material—including, where appropriate, alternative analyses conducted using distributional Bayesian models. Demographic data is extracted from participants’ Prolific profiles and used to control for age, sex, and ethnicity in all analyses.

Replication data, code, and a full survey transcript are available on OSF: https://osf.io/8qn4r/.

## Results

### Optimism, Hope, and Prospective Evaluations (H_1_)

**Fig. 2:**
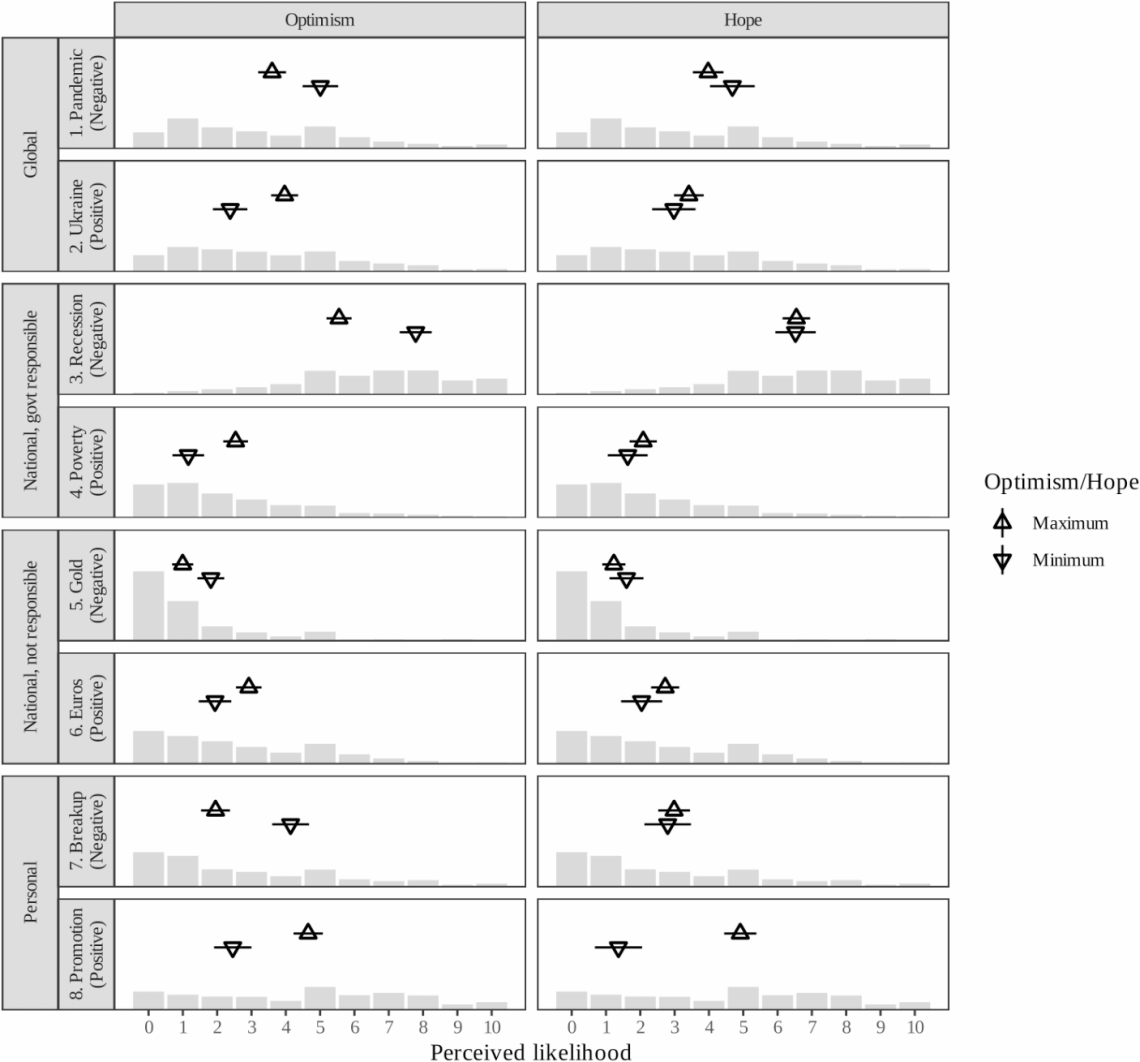
Model predictions of perceived likelihood of collective and individual outcomes, with 95% CIs, at maximal and minimal levels of optimism (full scale) and hope (full scale), plotted over raw distributions of responses for each outcome

To assess H_1_, Fig. 2 summarises the effects of optimism and hope on each of our first set of valence expectations items. Triangular points display the predicted likelihood rating assigned by the most or least optimistic/hopeful, and horizontal bars display the 95% confidence interval of this prediction, taken from models regressing the relevant outcome on both hope and optimism, with controls for age, gender, ethnicity, and party preference. The bars in each panel also display the raw, overall distribution of likelihood ratings assigned by our full sample to each of the possible future scenarios.

In every case, the effect of optimism is statistically significant. For example, the most optimistic respondents rate the chances of another pandemic approximately 1.4 points lower ($\beta =$ − 1.41, 95% CI: − 2.14, − 0.67), and the chance of a peaceful resolution to the war in Ukraine approximately 1.6 points higher ($\beta =$ 1.59, 95% CI: 0.87, 2.30), compared to the most pessimistic respondents. Similarly, optimists are significantly less likely to predict another recession ($\beta =$ −2.23, 95% CI: − 2.90, − 1.57) and significantly more likely to predict rates of poverty to improve ($\beta =$ 1.38, 95% CI: 0.72, 2.03).^7^ These results lend support to H_1OPT_. In comparison, the effects of optimism on the national, non-political outcomes are rather smaller. This may reflect such outcomes—especially in the sporting arena—being less subject to optimism. It may, however, reflect specific quirks with these items: a floor effect on the Olympic item, since even the most pessimistic respondents deemed it unlikely that Team GB would win no gold medals; and a biasing effect on the Euro football item courtesy of those from Scotland or Wales for whom optimism might point against an England victory! By contrast, large effects are observed at the personal level: optimists rate their chances of undergoing a breakup around 2.2 points lower ($\beta =$ − 2.19, 95% CI: − 2.95, − 1.42), and their chances of getting a promotion 2.2 points higher ($\beta =$ 2.20, 95% CI: 1.42, 2.97), than pessimists. However, neither effect is larger than that of optimism on expectations of a future recession, suggesting that while optimism may begin at home—it certainly can extend to matters of collective socio-political interest. Along with these effect sizes, visualised as the horizontal distance between each triangular point, Fig. 2 also shows how positivity and negativity varied across the items, net of any effect of optimism. For example, even the most optimistic are predicted to assign a relatively high likelihood to a future recession ($\widehat{y}=$ 5.55, 95% CI: 5.18, 5.92), and a relatively low likelihood to a reduction in poverty rates ($\widehat{y}=$ 2.54, 95% CI: 2.18, 2.90).^10^ However, there are not any striking or consistent patterns suggesting that people are generally positive or negative about the future.

By contrast, hope typically has no discernible effect on prospective evaluations. For all global and national outcomes, and for the perceived likelihood of undergoing a breakup, dispositionally hopeful people are not significantly more or less likely to foretell the outcome. In the right-hand panel of Fig. 2, the model-predicted likelihood ratings for the most and least hopeful barely differ, and their confidence intervals overlap substantially in almost every case. These findings strongly contradict H_1HOPE_. Where hope does have a significant and very large effect is on respondents’ estimated chances of getting a promotion ($\beta$ = 3.55, 95% CI: 2.56, 4.54). This result tallies with the agency-based definition of hope in that, of all the outcomes, promotion is the one where one’s own capacities are most influential over the result. (On this reading, however, respondents must take a discouragingly passive view of their close personal relationships.)

**Fig. 3:**
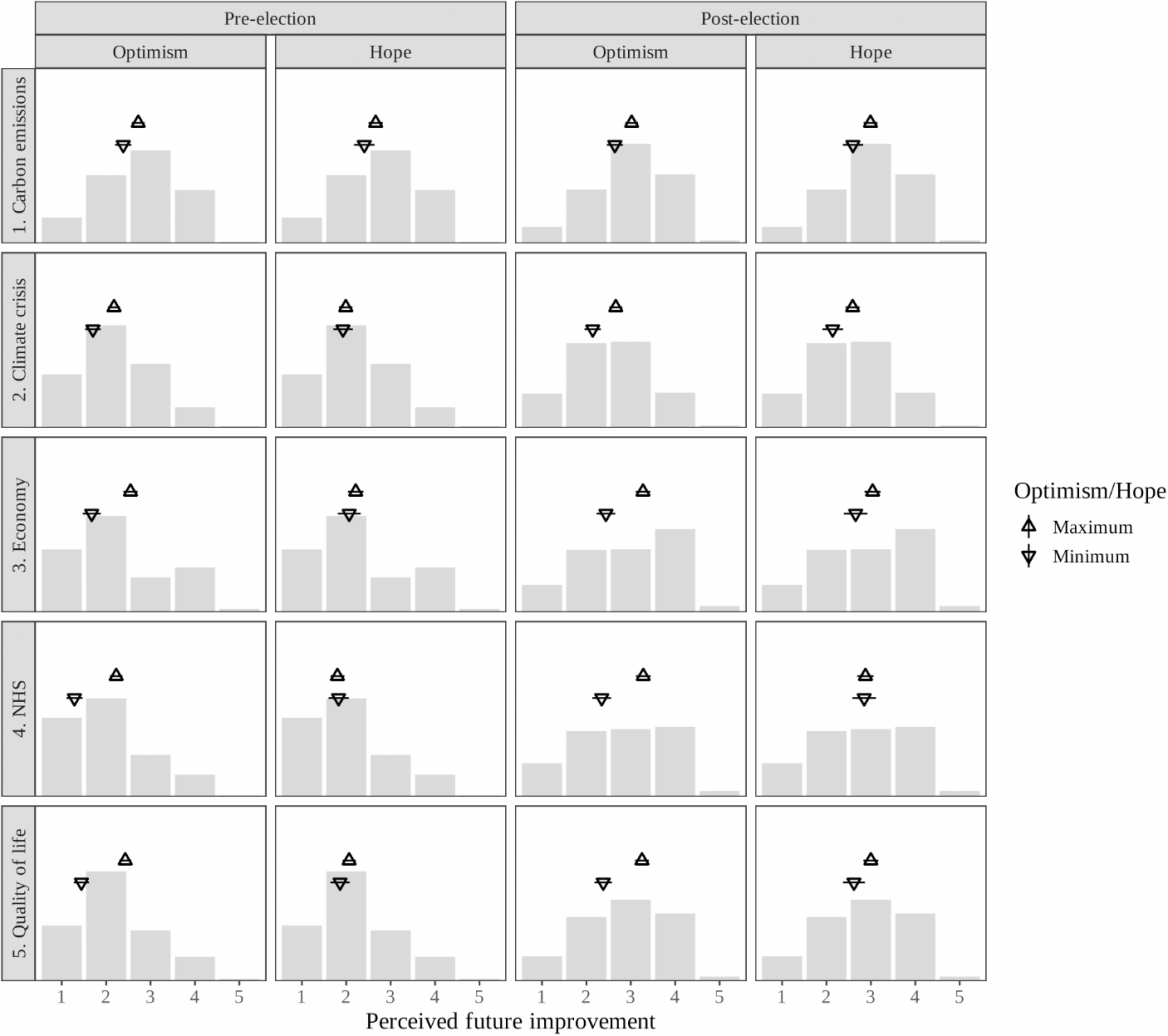
Model predictions of perceived likely future improvement in societal outcomes before and after the election, with 95% CIs, at maximal and minimal levels of optimism (full scale) and hope (full scale), plotted over raw distributions of responses for each outcome

Figure 3 displays the effects of optimism and hope on our second measure of valence expectations: prospective evaluation-type questions about whether a range of societal outcomes will get better or worse before and after the next election. The specification and approach to visualisation are the same as in the Fig. 2 models. Again, we find consistently significant effects of optimism. Hope, by contrast, is only significantly associated with more positive evaluations in three cases: perceptions that carbon emissions, the climate crisis, and quality of life will improve *after* the forthcoming election.^8^ These few *post-election* effects of hope could be a result of hopeful party supporters’ tendency to expect their preferred party to win the upcoming election (see below) and the assumption that this victory will produce societal benefits.

Indeed, net of any effect of optimism or hope, the distributions in Fig. 3 also suggest that there may be greater overall positivity about the chance of improvements after the election, than before. However, in most cases, regardless of levels of optimism or hope, people are more likely to expect things to get worse than get better. Negligible numbers of respondents reported expecting any of the outcomes to get ‘a lot’ better, whether before or after the election.

### Optimism, Hope, and Partisan Electoral Expectations (H_2–4_)

**Fig. 4:**
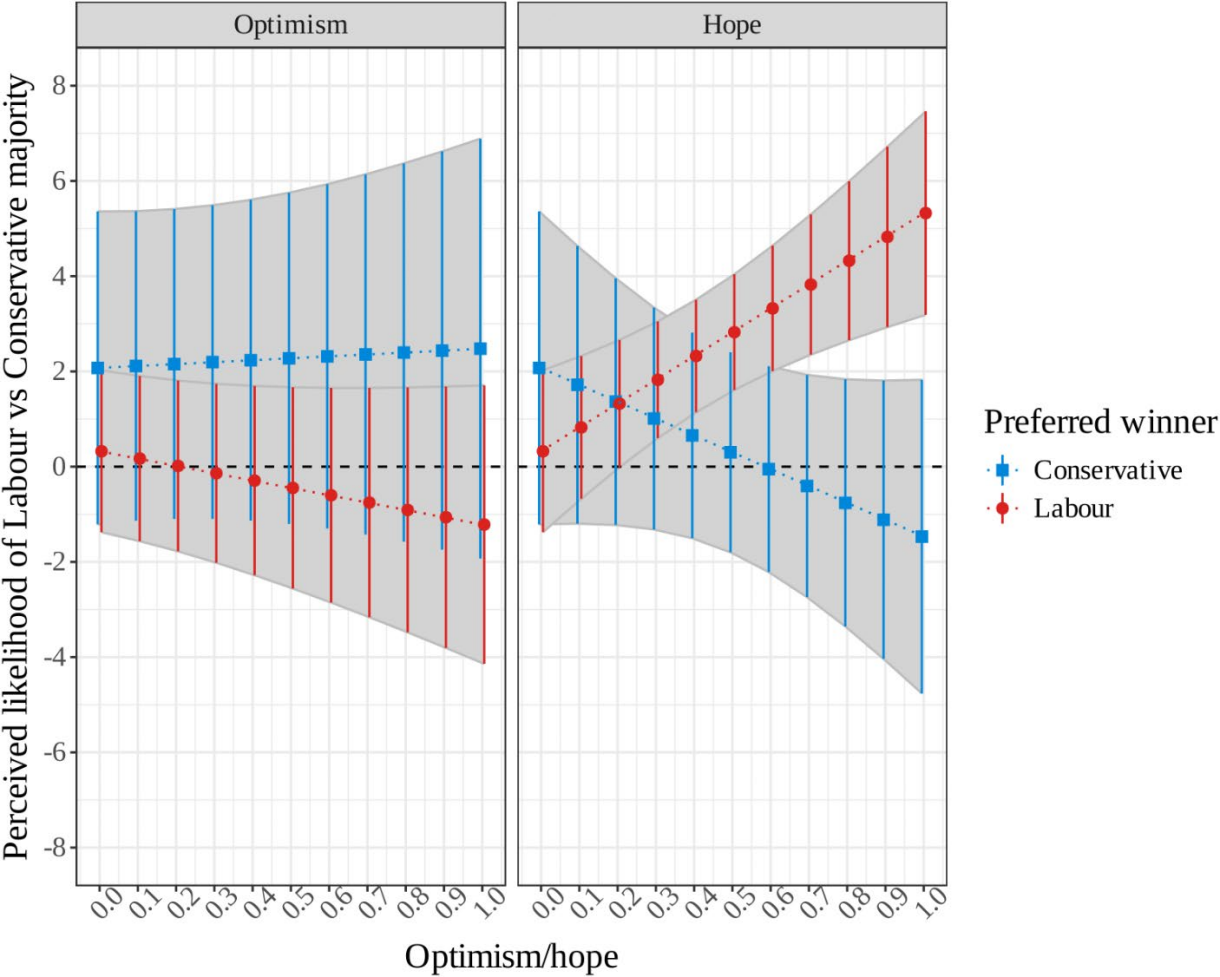
Predicted difference (and 95% CI) between perceived likelihood of Labour majority and perceived likelihood of Conservative majority, between Labour and Conservative supporters, by levels optimism and hope, in control group only

We now move from valence outcomes, desired across society, to electoral outcomes, where individuals differ markedly in their preferences. Before turning to the experimental analysis, we begin by looking at the interactive effect of hope or optimism and party preferences on expectations among the control group, who provide an unmanipulated measure of electoral expectations. Figure 4 displays the model-predicted differences in perceived likelihood of Labour and Conservative majority between those in the control group who want the Labour Party to win and those who want the Conservative Party to win, across levels of optimism and hope. For example, in the left panel, the blue points (and bars) display Conservative supporters’ perceptions of the difference between Labour and the Conservatives’ chance of forming a majority, across the range of dispositional optimism; in the right panel, the red points (and bars) display Labour supporters’ probability of selecting a Labour government (whether a majority or in coalition) as the most likely outcome, across the range of trait hope. In all cases, a larger positive value on the y-axis indicates a stronger perception that the Labour Party will beat the Conservative Party.

The results reveal no sign of a significant association between optimism and partisan expectations. Across the full range of optimism, partisans are indistinguishable in their perception of Labour’s advantage over the Conservative Party, corresponding to a null main effect of optimism on expectations ($\beta =$ 0.40, 95% CI: − 3.31, 3.89), and a null interaction effect between optimism and party preference ($\beta =$ − 1.94, 95% CI: − 6.05, 2.16). Similarly, Labour and Conservative supporters of all levels of optimism are statistically indistinguishable in their probability of choosing a Labour government as the most likely election outcome. Hope, however, tells a different story. Figure 4 shows that, at the lowest levels of hope, supporters of both parties are agreed that a Labour majority is slightly more likely than a Conservative one. At higher levels of hope, party supporters diverge in their expectations, reflecting a significant interaction effect ($\beta =$ 8.54, 95% CI: 2.64, 14.44). The most hopeful Labour supporters are predicted to rate its chances of forming a majority around 5.3 points higher ($\widehat{y}=$ 5.32, 95% CI: 3.18, 7.46) than the Conservative Party’s chances on the 0–10 scale, while the most hopeful Conservative supporters see barely any difference in the parties’ chances—if anything, marginally favouring a Conservative majority ($\widehat{y}=$ − 1.47, 95% CI: − 4.76, 1.82). We demonstrate the robustness of these findings in the Supplementary Material, where we analyze our secondary measure of electoral expectations and show that at the lowest levels of hope, partisans agree that a Labour government is the most likely outcome, but there is again a significant interaction effect. The most hopeful Labour supporters are overwhelmingly more likely to select a Labour government as most likely, while the most hopeful Conservative supporters are about as likely to select a Conservative government as a Labour government. Optimism has a null main and interaction effect on this alternative measure of electoral expectations.

These findings provide clear support for H_2HOPE_ and cast substantial doubt on H_2OPT_. Contrary to valence expectations, hope dominates optimism in the case of electoral expectations. The partisan bias so familiar from research on electoral expectations is actually manifest only among more *hopeful* partisans—but there it is strikingly powerful. Given that this analysis was confined to the control group, there was limited power to detect the kind of interactions plotted in Fig. 4. The same pattern emerges with greater precision when looking at the full sample, controlling for treatment status, as shown in the Supplementary Material.

Broadly speaking, there are two ways that the effects of hope observed in Fig. 4 might come about. One is that hope simply provides a boost to in-party expectations, regardless of the political context or information environment. Another is that hope shapes processing of that information: the same signal about a party’s prospects is interpreted more positively by its hopeful than its unhopeful partisans and thus, as information about an upcoming election flows in, their expectations diverge. The latter is the mechanism tested—for both hope and optimism—in our experiment.

**Fig. 5:**
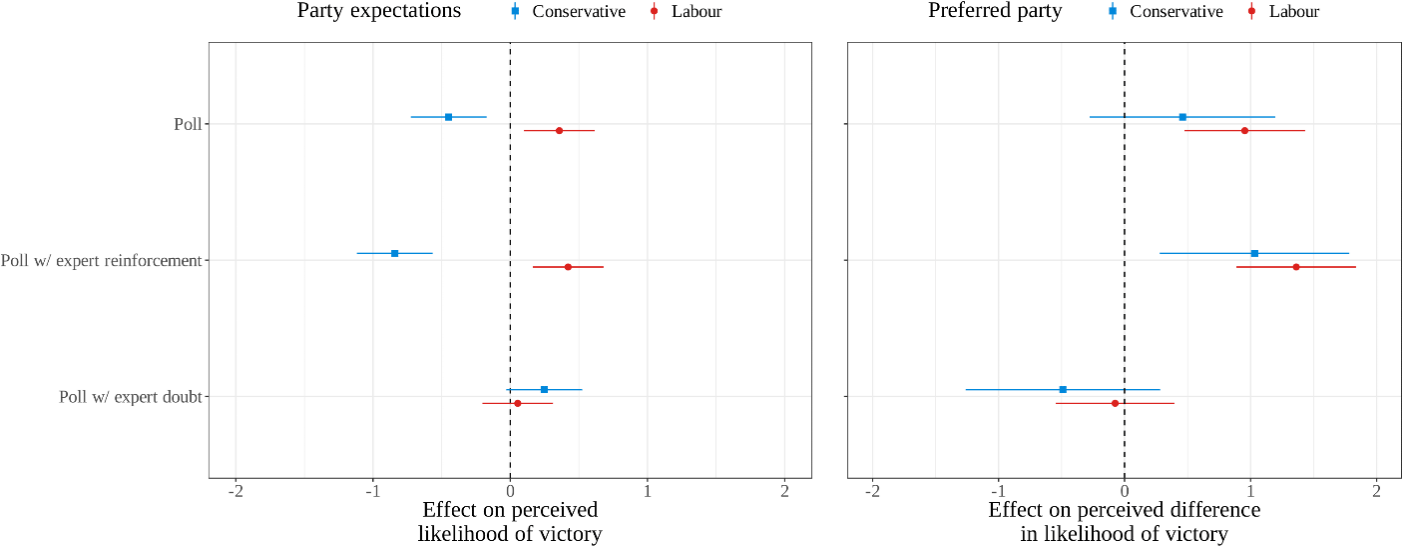
Effects (and 95% CI) of treatment conditions on electoral expectations, versus pure control condition. Left: average treatment effects on expectations for each party, controlling for age, gender, ethnicity, and preferred winner. Right: marginal treatment effects from interaction of perceived difference in likelihood of victory (likelihood of Labour victory minus likelihood of Conservative victory) by party preference, controlling for age, gender, and ethnicity

First, we verify that the treatments had a main effect on electoral expectations. Figure 5 confirms this effect, whether the outcome variable is the likelihood of each party winning (all respondents, left panel), or the difference in likelihood of a Labour win minus the likelihood of a Conservative win (by party preference, central panel). Both the poll treatment and especially the poll combined with expert reinforcement treatment significantly increase average expectations for the Labour Party ($\beta =$ 0.36, 95% CI: 0.10, 0.61; $\beta =$ 0.42, 95% CI: 0.16, 0.68) and reduce average expectations for the Conservative Party ($\beta =$ − 0.45, 95% CI: − 0.73, − 0.17; $\beta =$ − 0.84, 95% CI: − 1.12, − 0.57). While the right panel shows a degree of resistance among Conservative supporters when it comes to the poll-only treatment, generally there is consistent evidence that the first two treatments proved persuasive. Compared to the control group, these treatments shifted those on both sides of the partisan aisle in the direction of seeing Labour’s prospects as rosier relative to those of the Conservatives. Overall, these effects provide support for H_3_ and H_4_. We reinforce the robustness of these findings in the Supplementary Material, showing both treatments significantly affect our secondary measure of electoral expectations, increasing the probability that supporters of both Labour and the Conservatives would select a Labour government as the most likely outcome.

The poll combined with expert doubt treatment had null effects on expectations for both parties (left panel) and that overall null finding applies to partisans on both sides (right panel). The expert statement questioning the prediction of the poll cancels out its in-boosting effect on Labour supporters’ expectations and its out-boosting effect on Conservative supporters’ expectations. More generally, as the overlapping confidence intervals in the right-hand panel imply, the interaction effect between each treatment and party preferences is statistically insignificant. In short, preferences alone have little conditioning effect on the interpretation of this polling information.

**Fig. 6:**
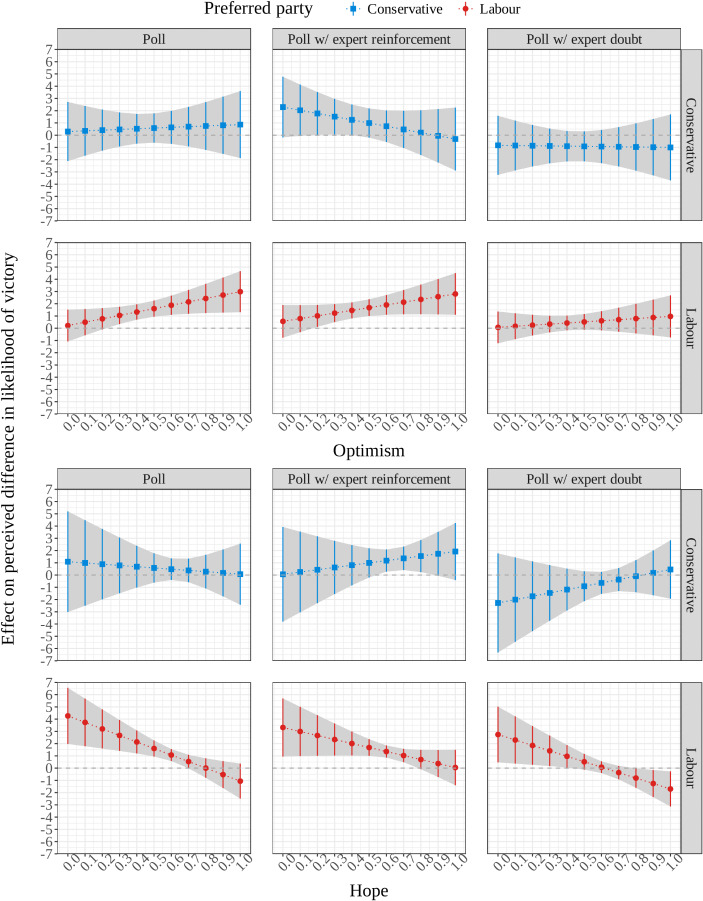
Effects (and 95% CIs) of treatment conditions on perceived electoral advantage of Labour party, versus pure control condition, by levels of optimism/hope, controlling for age, gender, and ethnicity

But how about preferences when galvanized by optimism or hope? Fig. 6 displays the marginal effects of each treatment, interacted with optimism and hope, on perceptions of the difference in the parties’ chances of forming a majority, among Conservative and Labour preferrers separately. Here, we again recode the dependent variable so that it measures the perceived likelihood that the Labour party will form a majority minus the likelihood that the Conservative party will do so. In line with what we observed in Fig. 5, we expect mostly positive marginal effects of the Poll and Poll with expert reinforcement treatments for both groups, and either null marginal effects, or a range of both positive and negative marginal effects, for the Poll with expert doubt treatment, for both groups. However, our primary interest lies in how these marginal effects vary across levels of optimism and hope, indicated by the slope between the points.

For Conservative supporters, the effects of treatment on the perceived electoral advantage of the Labour Party do not change significantly along the hope scale, but for Labour supporters, there is a negative interaction effect for both the poll treatment ($\beta =$ − 5.33, 95% CI: − 8.92, − 1.73) and for the poll combined with expert doubt ($\beta =$ − 4.44, 95% CI: − 8.04, − 0.85). The poll treatment ($\beta =$ 2.88, 95% CI: 1.00, 4.76) and the poll with expert doubt ($\beta =$ 2.29, 95% CI: 0.50, 4.08) both significantly raise expectations for the least hopeful Labour supporters, but this effect shrinks at higher levels of hope—even changing sign among the most hopeful Labour supporters in the expert doubt condition. The effect of the poll with expert reinforcement ($\beta =$ 2.20, 95% CI: 0.23, 4.17) is similar in size among the least hopeful Labour supporters, and shrinks to non-significance among the most hopeful, but this change in the effect across the hope scale is itself only significant at the 10% level ($\beta =$ − 3.27, 95% CI: − 6.97, 0.425).

These results indicate that the interaction between hope and electoral cues works in the opposite direction to what H_3HOPE_ and H_4HOPE_ suggest, and that this unanticipated negative relationship is driven primarily by supporters of the party for whom those cues are generally more favorable. Rather than suggesting that it is more hopeful partisans reading more (less) into positive (negative) predictive cues that produces partisan differences in expectations, our results suggest that those differences may be consolidated when exposure to such information brings less hopeful supporters more in line with their more hopeful co-partisans, whose expectations are already very high. Positive predictive cues give unhopeful partisans something to hope for. Indeed, the association between hope, party preferences and electoral expectations is so strong at baseline (as shown in Fig. 4 above) that the expectations of the most hopeful may even be subject to a ceiling effect, meaning the treatment can only raise the expectations of the least hopeful.

This pattern is robust to, and arguably even emerges more convincingly in, alternative specifications addressed in the Supplementary Material. Hope continues to negatively interact with treatment when pooling across treatments (comparing all treatment groups combined against the control group), and when pooling across electoral preferences (grouping together those who prefer a Labour and a Conservative victory). Hope also significantly moderates all three treatments’ effects on our secondary measure of electoral expectations—choosing a Labour government as the most likely outcome.

There is, however, a hint of support for H_3OPT_: the effect of the poll treatment is significantly stronger among optimistic Labour supporters ($\beta =$ 2.77, 95% CI: 0.07, 5.47). But this effect is only marginally significant (*p* = 0.045), and the interaction effect between optimism and treatment is non-significant in all other cases. This positive interaction effect is also not robust to those alternative specifications, reported in the Supplementary Material, to which we just noted the negative interaction effect of hope was robust. Overall, there is therefore little support for H_3OPT_ and no support for H_4OPT_. Generally speaking, the effect of a poll, with or without an expert in-boost, is more or less the same regardless of a respondent’s level of dispositional optimism.

## Discussion

Tikochinski and Babad (2023, 252) recently commented on ‘wishful thinking’ research that ‘the field is rather quiet, with relatively few new studies, no sweeping theoretical innovations and no dramatic debates’. In this paper, we have presented a ‘new study’ in the field of political expectations and introduced a ‘theoretical innovation’ in the form of the distinction between hope and optimism. Furthermore, while ‘dramatic debate’ may overstate the case, our findings do make significant novel contributions to understanding the origins of expectations. We highlight optimism as the key driver of valence expectations but hope as the engine of electoral expectations. In other words, partisan expectations at election time do not seem to represent blind faith in a positive outcome. Rather, people may believe their party can win because they conceive this outcome as a goal that their side can achieve. This clarifies the nature of ‘wishful thinking’ involved and should deter political scientists from equating it with optimism.

Accordingly, we also highlight a striking curb on such ‘wishful thinking’. There was no tendency to express particularly partisan electoral expectations among partisans scoring low on the hope scale. It takes a *combination* of partisan goals and hope to generate this effect. Since hope is goal-orientated, this mechanism suggests that one strand of people’s identification with, or support for, parties is to take those parties’ goals as their own. That echoes broader social psychological work showing the role of common goals and concerns in forging group identity (Hogg and Reid 2006).

Since less hopeful partisans were not prone to generate their own positive expectations of electoral victory, it makes sense that they were more in need of—and hence more responsive to—the positivity provided in our informational treatment. The hopeful needed no persuading; the less hopeful could be talked into positivity. This may explain how rival partisans come to hold such clearly divergent beliefs about likely election outcomes. Predictive cues from polls and experts—the most important feature of the media environment in this context—contribute to bringing the expectations of unhopeful supporters in line with their more hopeful co-partisans, pulling the expectations of partisan groups further apart from each other on average. Social networks are likely to play a role here, too (see Leiter et al. 2018; Mongrain 2021). Party allegiances tend to shape the formation of political discussion networks and, if partisans then drift into using these fairly homogeneous networks as the basis for judging public opinion, this will further skew their electoral expectations—regardless of whether they score lower on dispositional hope.

Moving from hope to optimism, and taking public opinion in the aggregate, there is a contrast between people’s general tendency to optimism about the future (e.g. Baranski et al. 2021) and the tendency to pessimism that often characterises expectations in the economic and political realms. However, any conclusion that optimism simply does not extend to politics is contradicted by our results at the individual level. While the intercept may be lower, with citizens generally struggling to find grounds for confidence in the political future, the slope is still clear: those dispositionally more optimistic are also clearly and consistently more positive than pessimists—across a range of political issues, measures, and time frames. We have tended towards interpreting this consistent association as showing a causal effect of dispositional optimism on societal valence expectations. However, there is an alternative interpretation, namely that our measures of valence expectations are really *indicators* of the latent construct of optimism rather than *consequences* of it. The two are only subtly different conceptually, and they cannot easily be separated empirically with the given data. But we would note that the measure of dispositional optimism was not an index of *specific* positive forecasts, such as those in our valence expectations battery. The items on that scale instead make general references, e.g. to ‘good things’ or to what respondents ‘usually expect’. Perhaps a compromise interpretation is to see the valence expectations as specific applications of the general predisposition to optimism. In any event, it is clear that politics is a domain in which optimism can and does apply.

It bears emphasising that our results were obtained from models in which the other disposition was held constant. While this was the right specification to address our hypotheses, it does shape the interpretation of the coefficients. Since the key conceptual differences between hope and optimism are the senses of personal salience and agency associated with hope, the effect of hope on electoral expectations might be read as the effect of those senses, while the effect of optimism on valence expectations might demonstrate the lack of any such sense when considering the valenced societal future. It seems that citizens feel that they can contribute to electoral victory but not to economic growth, for example. Our findings thus have the further benefit of providing clues as to the aspects of politics where citizens feel some sense of agency and those where they feel they have less of a say. In particular, they may highlight party politics as generating not only group identities but also achievable purpose. In a narrow sense, this might be dismissed as irrational given the vanishingly small probability that any individual voter, however strongly partisan, can affect an election outcome. But partisanship is a collective identity and thereby an arena of “yes we can”, not “yes I can”. On this account, hope provides a possible resolution to the paradox of voting—and other forms of collective action. Some people are simply more predisposed to believe that their goals are achievable. These people seem highly likely to be overrepresented among political activists across the board. That hypothesis can and should be tested in future research.

## Supplementary Information

Below is the link to the electronic supplementary material.

Supplementary file1 (PDF 707 kb)

## Data Availability

Replication data, code, and a full survey transcript are available on OSF: https://osf.io/8qn4r/.

## Footnotes

1 Pessimism is the tendency to believe that bad outcomes are likely to come about. There is debate over whether optimism and pessimism are polar opposites or two distinct traits. We do not distinguish them conceptually here. In our analyses below we employ an overall, bipolar optimism–pessimism scale. As discussed in the results section below, we report the results using decomposed scales in our Supplementary Material, following Carver and Scheier’s (2014, 294) recommendations.

2 Note that our hypotheses are reordered and reworded relative to how they appear in our pre-registration (https://osf.io/9tb4d/?view_only=628fd097d085447cb3adfe67e252ed94), purely for expositional purposes. They retain the same meaning. For full transparency, the Supplementary Material matches each hypothesis across the two documents. We also pre-registered a further hypothesis regarding the effects of optimism and hope on ‘affective forecasts’. We address this hypothesis only in the Supplementary Material.

3 To generate a representative sample, Prolific divides the requested sample size into age, sex and ethnicity subgroups that mirror the proportions of the national population, based on census data. The study is made available to eligible respondents who complete the survey on a first-come, first-served basis, according to their satisfaction of these demographic criteria. For example, once the required number of 28–37-year-old white men has been recruited to reflect their proportion in the national population in the complete sample, 28–37-year-old white men are no longer eligible to complete the study. See: https://researcher-help.prolific.com/en/article/e6555f.

4 Actual median completion time was 6 min 21 s, yielding an average payment of £11.34/hour.

5 As pre-registered, we replaced the scale’s filler items with four items from the Psychological Preparedness for Disaster Threat Scale (excluded here), as measures of preparedness (McLennan, Marques, and Every 2020). We discuss this choice and assess the relationship of this preparedness subscale to our key dependent variables in the Supplementary Material, where we also list its items.

6 Professor Jennings’ thread was a response to Professor Ford’s.

7 In the Supplementary Material, we find that these results are generally consistent when measuring optimism only using the positively worded items in the scale, and observe mostly significant inverse effects for pessimism, measured using only the negatively worded items in the scale (Carver and Scheier 2014, 294). However, when entering both subscales as predictors into the same model, in most cases, only optimism has a significant effect on expectations of a positive outcome, and only pessimism has a significant effect on expectations of negative outcomes (like a recession). The only case for which this pattern does not hold is personal-level expectations.

10 In generating these predicted values, we hold all control variables at their baseline levels, including notably party preference, which is fixed as Labour. 

8 In the Supplementary Material, we apply Bayesian ordinal regression models that are better suited to the analysis of likert outcome data (Bürkner and Vuorre 2019) finding further, strong support for the effect of optimism, and further suggestion that hope might have a small effect on prospective post-election evaluations.
