# Supplementary material for: Hope, Optimism, and Expectations for the Political Future
Source: Polit Behav. 2025 Mar 13;48(1):205–28. doi: 10.1007/s11109-025-10027-5 (PMC12904912; doi:10.1007/s11109-025-10027-5)
Supplement: Supplementary file 1 — Supplementary file1 (PDF 707 kb) [file 11109_2025_10027_MOESM1_ESM.pdf]

Supplementary Material: Hope, Optimism, and Expectations in Politics

Contents

1 Hypothesis disambiguation **2**

2 Optimism and hope scales **2**
- 2.1 Adapted Life Orientation Test 2
- 2.2 Adapted Adult Hope Scale 3
- 2.3 Factor analyses 4

3 Valence expectations **9**
- 3.1 Distributions 9
- 3.2 Main model tables 12
- 3.3 Subscale effects 31
- 3.4 Bayesian models 31

4 Electoral expectations **38**
- 4.1 Distributions of expectations 38
- 4.2 Liberal Democrat expectations 38
- 4.3 Full sample interactions 42
- 4.4 Model summaries 42
- 4.5 Subscale effects 42
- 4.6 Majority/coalition items 48
- 4.7 Pooled analyses 54
- 4.8 Outperform predictions items 55

5 Affective forecasts **56**
- 5.1 Additional hypotheses 56
- 5.2 Results 57

6 Preparedness **58**
- 6.1 Exploratory hypotheses 58
- 6.2 Results 60

References **63**

1 Hypothesis disambiguation

Table 1: Correspondence between pre-registered hypotheses and hypotheses in manuscript/supplementary material.

Preregistration	Manuscript
H1	H1HOPT and H1HOPE
H2	H2HOPT and H2HOPE
H3a	H3OPT and H3HOPE
H3b	H6OPT and H6HOPE
H4a	H4
H4b	H4OPT and H4HOPE
H5a	H5
H5b	H5OPT and H5HOPE

2 Optimism and hope scales

2.1 Adapted Life Orientation Test

All items measured on 0-4 scale ranging from strongly disagree to strongly agree:

- In uncertain times, I usually expect the best (positively coded/optimism subscale)
- It's easy for me to relax (filler item)
- If something can go wrong for me, it will (negatively coded/pessimism subscale)
- I'm always optimistic about my future (positively coded/optimism subscale)

- I enjoy my friends a lot (filler item)
- It's important for me to keep busy (filler item)
- I hardly ever expect things to go my way (negatively coded/pessimism subscale)
- I don't get upset too easily (filler item)
- I rarely count on good things happening to me (negatively coded/pessimism subscale)
- Overall, I expect more good things to happen to me than bad (positively coded/optimism subscale)
- This year will be better than last year for me (positively coded/optimism subscale) (discretionary item removed following initial factor analysis)
- I get nervous easily (filler item)

2.2 Adapted Adult Hope Scale

All items measured on 0-4 scale ranging from strongly disagree to strongly agree. Some items are included from Psychological Preparedness for Disaster Threat Scale, replacing filler items:

- I can think of many ways to get out of a jam (pathway subscale)
- I energetically pursue my goals (agency subscale)
- I am able to manage my feelings pretty well in difficult and challenging situations (preparedness)
- There are lots of ways around any problem (pathway subscale)
- I have a good idea of how I would likely respond in an emergency (preparedness)
- I can think of many ways to get the things in life that are important to me (pathway subscale)
- I feel reasonably confident in my ability to deal with stressful situations that I might find myself in (preparedness)
- Even when others get discouraged, I know I can find a way to solve the problem (pathway subscale)

- My past experiences have prepared me well for my future (agency subscale)
- I've been pretty successful in life (agency subscale)
- I seem able to stay cool and calm in most difficult moments (preparedness)
- I meet the goals that I set for myself (agency subscale)

2.3 Factor analyses

Tables 2-5 display the fit statistics and factor loadings for increasingly complex factor analyses. Table 2 shows the results of a specification in which all optimism and hope items constitute one single construct. This model has poor fit. Table 3 shows the results of a specification in which all optimism items constitute one construct and all hope items constitute one second construct. This model has acceptable fit and informs the analyses in the main paper. Fit is improved further by separating hope into agency and pathway constructs, as shown by Table 4. Fit is improved further still by distinguishing optimism and pessimism constructs, by separating positively coded and negatively coded optimism items, as shown by Table 5. In our 'Subscale effects' analyses below we find that separating out the scales does not significantly alter the conclusions drawn from our main findings.

Table 2: Single factor CFA shows poor fit (note: all loadings $p < 0.001$).

**Name**	**Value**
**Fit statistics**	
**CFI**	0.802
**TLI**	0.766
**RMSEA**	0.137
**SRMR**	0.083
**Factor loadings (optimism/hope)**	
**Optimism 1**	1.000
**Optimism 2**	0.960
**Optimism 3**	1.194
**Optimism 4**	1.119
**Optimism 5**	1.117
**Optimism 6**	1.089
**Agency 1**	0.875
**Agency 2**	0.744
**Agency 3**	0.996
**Agency 4**	0.811
**Pathway 1**	0.726
**Pathway 2**	0.609
**Pathway 3**	0.894
**Pathway 4**	0.772

Table 3: Two factor CFA shows acceptable fit (note: all loadings p < 0.001).

Name	Value
**Fit statistics**	
**CFI**	0.924
**TLI**	0.910
**RMSEA**	0.085
**SRMR**	0.051
**Factor loadings (optimism)**	
**Optimism 1**	1.000
**Optimism 2**	1.055
**Optimism 3**	1.130
**Optimism 4**	1.223
**Optimism 5**	1.208
**Optimism 6**	1.115
**Factor loadings (hope)**	
**Agency 1**	1.000
**Agency 2**	0.848
**Agency 3**	1.037
**Agency 4**	0.926
**Pathway 1**	0.885
**Pathway 2**	0.711
**Pathway 3**	1.021
**Pathway 4**	0.892

Table 4: Three factor CFA shows improved fit (note: all loadings $p < 0.001$).

Name	Value
**Fit statistics**	
**CFI**	0.935
**TLI**	0.921
**RMSEA**	0.080
**SRMR**	0.048
**Factor loadings (optimism)**	
**Optimism 1**	1.000
**Optimism 2**	1.056
**Optimism 3**	1.132
**Optimism 4**	1.225
**Optimism 5**	1.209
**Optimism 6**	1.115
**Factor loadings (agency hope)**	
**Agency 1**	1.000
**Agency 2**	0.821
**Agency 3**	1.042
**Agency 4**	0.936
**Factor loadings (pathway hope)**	
**Pathway 1**	1.000
**Pathway 2**	0.794
**Pathway 3**	1.086
**Pathway 4**	0.991

Table 5: Four factor CFA shows very good fit (note: all loadings $p < 0.001$).

**Name**	**Value**
**Fit statistics**	
**CFI**	0.966
**TLI**	0.956
**RMSEA**	0.059
**SRMR**	0.035
**Factor loadings (optimism)**	
**Optimism 1**	1.000
**Optimism 3**	1.155
**Optimism 6**	1.088
**Factor loadings (pessimism)**	
**Optimism 2**	1.000
**Optimism 4**	1.188
**Optimism 5**	1.123
**Factor loadings (agency hope)**	
**Agency 1**	1.000
**Agency 2**	0.818
**Agency 3**	1.038
**Agency 4**	0.932
**Factor loadings (pathway hope)**	
**Pathway 1**	1.000
**Pathway 2**	0.795
**Pathway 3**	1.092
**Pathway 4**	0.998

3 Valence expectations

3.1 Distributions

Figure 1 displays the distributions of responses to each of our global, national, and personal prospective evaluations items. There is clear variability in positivity and negativity across items. For example, at the national level, far more people think it likely than not that the UK will undergo another recession in the coming years, while the vast majority of respondents think it unlikely that rates of poverty will decrease. These observations potentially reveal a tendency towards negativity about national politics: a negatively valenced outcome is seen as likely whereas a positively valenced outcome is deemed unlikely. This pattern is not apparent in any other pair of outcomes. At the global level, while most people think it is unlikely that the war in Ukraine will be peacefully resolved any time soon, they are also more likely to report believing it unlikely that there will be another pandemic. Similarly, while the overwhelming majority of respondents think it highly unlikely that Team GB will fail to win any gold medals at the next Olympics, most also think it unlikely that England will win the Euros.[1] At the personal level, people mostly think it unlikely that they or someone close to them will experience a breakup soon, but the distribution of expectations about whether they or someone close to them will get a big promotion at work is fairly uniform.

Figure **??** displays the distributions of responses to our get better/worse prospectiv evaluations measures. Across most items, when asked about how the outcome will change before the next election, the modal response is that it will get a little worse, and respondents are more likely to expect the outcome to get worse than better. Asked about how things will change after the election, responses are much more

[1] Note, however, that this latter outcome may not have been desirable for respondents outside of England.

evenly spread across positive and negative changes. This pattern raises the possibility that voters might adjust their long-run expectations of societal outcomes based on their belief that their party will enter into government at the next opportunity.

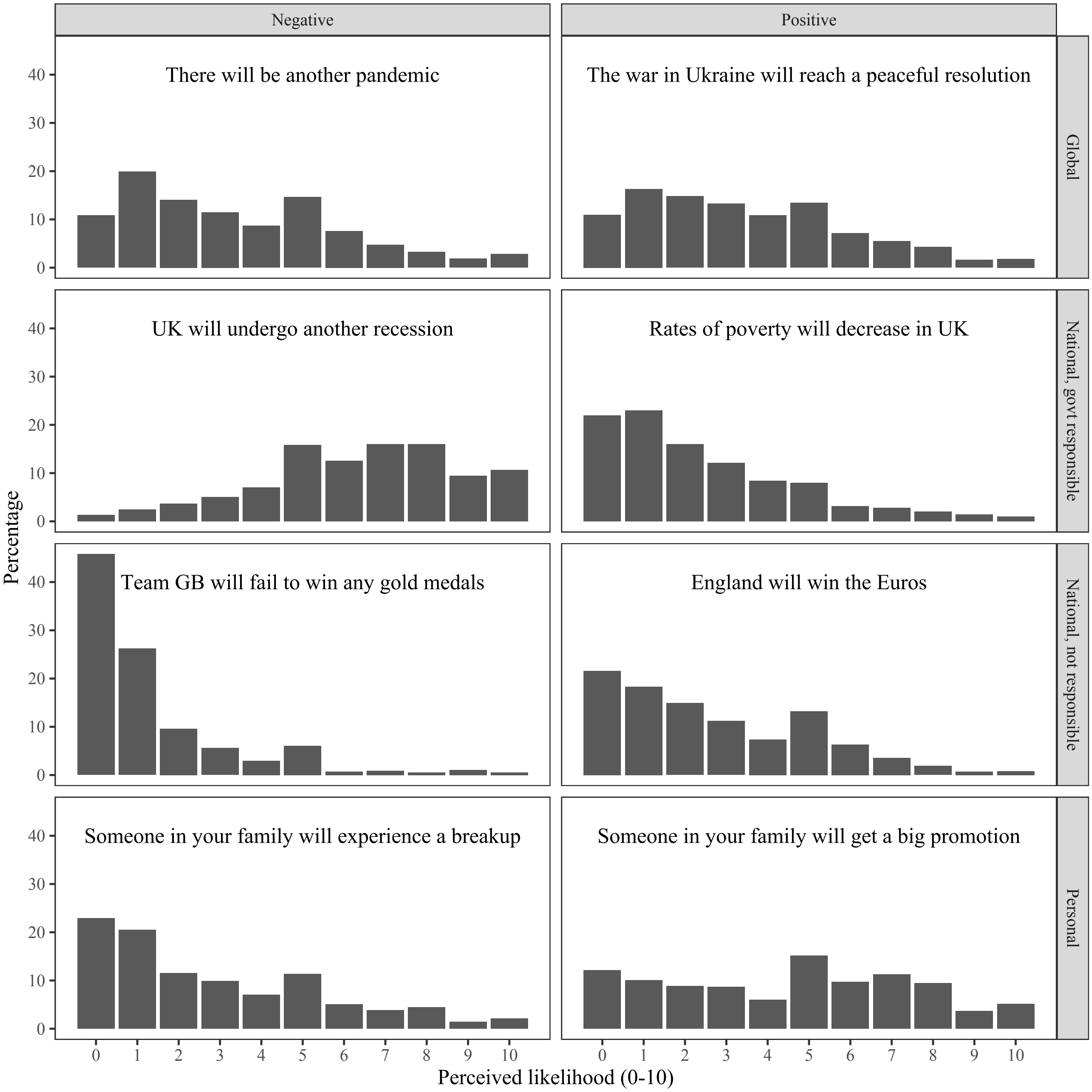

Figure 1: Distributions of responses to prospective evaluations items.

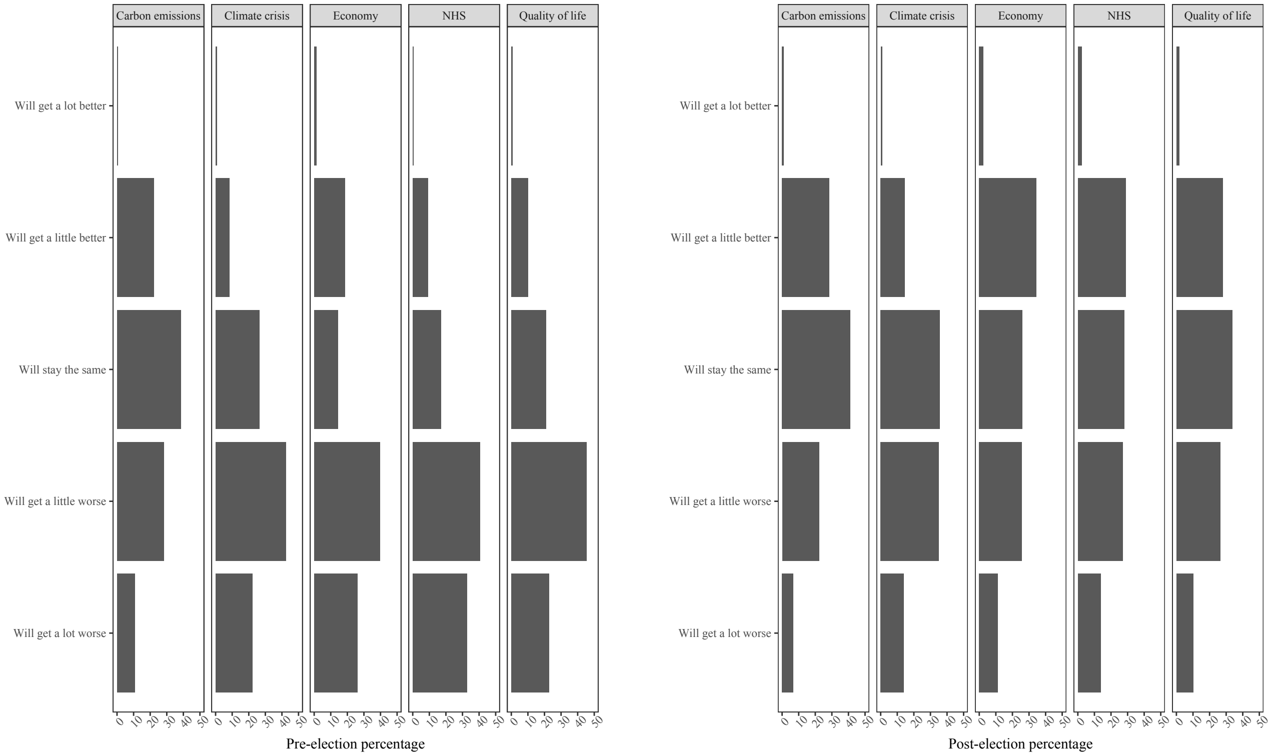

Figure 2: Distributions of responses to get better/worse prospective evaluations items.

3.2 Main model tables

Tables 6-23 provide full tabulated summaries of the prospective evaluations models reported in the main text, capturing the effects of optimism and hope on our prospective evaluations items while controlling for party preference, age, gender, and ethnicity.

Table 6: Full model summary, effect of optimism and hope on pandemic prospective evaluation.

	Dependent variable:
	There will be another pandemic
Intercept	3.755*
	(0.322)
Optimism	−1.406*
	(0.375)
Hope	−0.705
	(0.481)
Prefer Labour	0.322*
	(0.145)
Age 28-37	0.176
	(0.212)
Age 38-47	0.407
	(0.208)
Age 48-57	0.195
	(0.219)
Age 58+	1.076*
	(0.196)
Female	0.313*
	(0.124)
Asian	0.279
	(0.241)
Black	0.125
	(0.359)
Mixed	0.234
	(0.528)
Other ethnicity	0.633
	(0.685)
Observations	1,693
R^2	0.048
Adjusted R^2	0.041
Residual Std. Error	2.545 (df = 1680)
F Statistic	6.998* (df = 12; 1680)
Note:	$^{*}p < 0.05$

Table 7: Full model summary, effect of optimism and hope on Ukraine prospective evaluation.

	Dependent variable:
	The war in Ukraine will reach a peaceful resolution
Intercept	2.411*
	(0.312)
Optimism	1.587*
	(0.364)
Hope	0.437
	(0.466)
Prefer Labour	−0.376*
	(0.140)
Age 28-37	0.113
	(0.206)
Age 38-47	−0.031
	(0.201)
Age 48-57	0.313
	(0.212)
Age 58+	0.043
	(0.190)
Female	0.015
	(0.120)
Asian	0.462*
	(0.234)
Black	0.833*
	(0.348)
Mixed	0.244
	(0.512)
Other ethnicity	−0.473
	(0.665)
Observations	1,694
R^2	0.041
Adjusted R^2	0.034
Residual Std. Error	2.468 (df = 1681)
F Statistic	5.998* (df = 12; 1681)
Note:	$^{*}p < 0.05$

Table 8: Full model summary, effect of optimism and hope on recession prospective evaluation.

	Dependent variable:
	The UK will undergo another recession
Intercept	7.028*
	(0.290)
Optimism	−2.234*
	(0.338)
Hope	0.025
	(0.433)
Prefer Labour	1.012*
	(0.131)
Age 28-37	−0.074
	(0.192)
Age 38-47	0.084
	(0.187)
Age 48-57	−0.136
	(0.197)
Age 58+	−0.185
	(0.177)
Female	−0.086
	(0.112)
Asian	−0.038
	(0.218)
Black	−0.584
	(0.324)
Mixed	0.352
	(0.477)
Other ethnicity	−0.097
	(0.618)
Observations	1,694
R^2	0.095
Adjusted R^2	0.089
Residual Std. Error	2.295 (df = 1681)
F Statistic	14.774* (df = 12; 1681)
Note:	*$p < 0.05$

Table 9: Full model summary, effect of optimism and hope on poverty prospective evaluation.

	Dependent variable:
	Rates of poverty will decrease in the UK
Intercept	2.140*
	(0.287)
Optimism	1.378*
	(0.334)
Hope	0.451
	(0.428)
Prefer Labour	−0.718*
	(0.129)
Age 28-37	−0.078
	(0.189)
Age 38-47	0.080
	(0.185)
Age 48-57	−0.144
	(0.195)
Age 58+	−0.153
	(0.175)
Female	−0.404*
	(0.110)
Asian	0.343
	(0.215)
Black	0.340
	(0.320)
Mixed	−0.165
	(0.471)
Other ethnicity	−1.063
	(0.610)
Observations	1,693
R^2	0.057
Adjusted R^2	0.050
Residual Std. Error	2.266 (df = 1680)
F Statistic	8.410* (df = 12; 1680)
Note:	*$p < 0.05$

Table 10: Full model summary, effect of optimism and hope on gold medals prospective evaluation.

	Dependent variable:
	Team GB will win no gold medals at the 2024 Olympics
Intercept	1.991*
	(0.244)
Optimism	−0.819*
	(0.284)
Hope	−0.369
	(0.364)
Prefer Labour	0.078
	(0.110)
Age 28-37	−0.049
	(0.161)
Age 38-47	0.014
	(0.157)
Age 48-57	−0.289
	(0.166)
Age 58+	−0.148
	(0.149)
Female	0.134
	(0.094)
Asian	0.089
	(0.183)
Black	0.218
	(0.272)
Mixed	0.566
	(0.400)
Other ethnicity	−0.229
	(0.519)
Observations	1,693
R^2	0.021
Adjusted R^2	0.014
Residual Std. Error	1.929 (df = 1680)
F Statistic	2.987* (df = 12; 1680)
Note:	$^*p < 0.05$

Table 11: Full model summary, effect of optimism and hope on Euros prospective evaluation.

	Dependent variable:
	England will win the Euros
Intercept	1.944*
	(0.296)
Optimism	0.985*
	(0.345)
Hope	0.687
	(0.441)
Prefer Labour	0.053
	(0.133)
Age 28-37	−0.326
	(0.195)
Age 38-47	−0.340
	(0.191)
Age 48-57	−0.636*
	(0.201)
Age 58+	−0.727*
	(0.180)
Female	0.219
	(0.114)
Asian	0.591*
	(0.222)
Black	0.359
	(0.330)
Mixed	−0.088
	(0.485)
Other ethnicity	0.369
	(0.629)
Observations	1,694
R^2	0.035
Adjusted R^2	0.028
Residual Std. Error	2.337 (df = 1681)
F Statistic	5.063* (df = 12; 1681)
Note:	*$p < 0.05$

Table 12: Full model summary, effect of optimism and hope on breakup prospective evaluation.

	Dependent variable:
	You or someone in your family will experience a breakup
Intercept	3.945*
	(0.334)
Optimism	−2.187*
	(0.390)
Hope	0.187
	(0.499)
Prefer Labour	0.208
	(0.151)
Age 28-37	−0.334
	(0.221)
Age 38-47	−0.122
	(0.216)
Age 48-57	−0.219
	(0.227)
Age 58+	−0.220
	(0.204)
Female	0.084
	(0.129)
Asian	−0.369
	(0.251)
Black	0.090
	(0.373)
Mixed	0.881
	(0.549)
Other ethnicity	0.352
	(0.712)
Observations	1,694
R^2	0.038
Adjusted R^2	0.031
Residual Std. Error	2.644 (df = 1681)
F Statistic	5.490* (df = 12; 1681)
Note:	$^{*}p < 0.05$

Table 13: Full model summary, effect of optimism and hope on promotion prospective evaluation.

	Dependent variable:
	You or someone in your family will get a big promotion
Intercept	1.839*
	(0.339)
Optimism	2.198*
	(0.395)
Hope	3.550*
	(0.506)
Prefer Labour	0.038
	(0.153)
Age 28-37	−0.279
	(0.224)
Age 38-47	−1.035*
	(0.219)
Age 48-57	−1.705*
	(0.230)
Age 58+	−1.847*
	(0.206)
Female	0.108
	(0.130)
Asian	0.989*
	(0.254)
Black	1.988*
	(0.378)
Mixed	0.128
	(0.556)
Other ethnicity	0.080
	(0.722)
Observations	1,694
R^2	0.195
Adjusted R^2	0.190
Residual Std. Error	2.679 (df = 1681)
F Statistic	34.041* (df = 12; 1681)
Note:	*$p < 0.05$

Table 14: Full model summary, effect of optimism and hope on carbon emission pre-election prospective evaluation.

	Dependent variable:
	Pre-election: carbon emissions (will get much/slightly better/worse)
Intercept	2.655*
	(0.115)
Optimism	0.338*
	(0.134)
Hope	0.256
	(0.172)
Prefer Labour	−0.347*
	(0.052)
Age 28-37	0.022
	(0.076)
Age 38-47	0.099
	(0.074)
Age 48-57	0.157*
	(0.078)
Age 58+	0.139*
	(0.070)
Female	−0.229*
	(0.044)
Asian	−0.054
	(0.086)
Black	0.211
	(0.128)
Mixed	0.029
	(0.189)
Other ethnicity	0.481*
	(0.245)
Observations	1,694
R^2	0.072
Adjusted R^2	0.065
Residual Std. Error	0.909 (df = 1681)
F Statistic	10.839* (df = 12; 1681)
Note:	$^{*}p < 0.05$

Table 15: Full model summary, effect of optimism and hope on climate crisis pre-election prospective evaluation.

	Dependent variable:
	Pre-election: climate crisis (will get much/slightly better/worse)
Intercept	2.375*
	(0.109)
Optimism	0.475*
	(0.127)
Hope	0.062
	(0.162)
Prefer Labour	−0.580*
	(0.049)
Age 28-37	0.025
	(0.072)
Age 38-47	0.130
	(0.070)
Age 48-57	0.008
	(0.074)
Age 58+	−0.015
	(0.066)
Female	−0.119*
	(0.042)
Asian	−0.099
	(0.082)
Black	0.400*
	(0.121)
Mixed	−0.050
	(0.179)
Other ethnicity	0.269
	(0.232)
Observations	1,694
R^2	0.113
Adjusted R^2	0.107
Residual Std. Error	0.860 (df = 1681)
F Statistic	17.894* (df = 12; 1681)
Note:	$^{*}p < 0.05$

Table 16: Full model summary, effect of optimism and hope on economy pre-election prospective evaluation.

	Dependent variable:
	Pre-election: economy (will get much/slightly better/worse)
Intercept	2.130*
	(0.127)
Optimism	0.874*
	(0.148)
Hope	0.147
	(0.190)
Prefer Labour	−0.602*
	(0.057)
Age 28-37	−0.021
	(0.084)
Age 38-47	0.085
	(0.082)
Age 48-57	0.104
	(0.086)
Age 58+	0.226*
	(0.077)
Female	−0.175*
	(0.049)
Asian	−0.078
	(0.095)
Black	0.543*
	(0.142)
Mixed	−0.219
	(0.209)
Other ethnicity	−0.053
	(0.271)
Observations	1,694
R^2	0.148
Adjusted R^2	0.142
Residual Std. Error	1.005 (df = 1681)
F Statistic	24.349* (df = 12; 1681)
Note:	*$p < 0.05$

Table 17: Full model summary, effect of optimism and hope on NHS pre-election prospective evaluation.

	Dependent variable:
	Pre-election: NHS (will get much/slightly better/worse)
Intercept	2.015*
	(0.113)
Optimism	0.941*
	(0.132)
Hope	−0.027
	(0.169)
Prefer Labour	−0.493*
	(0.051)
Age 28-37	−0.052
	(0.075)
Age 38-47	−0.056
	(0.073)
Age 48-57	−0.077
	(0.077)
Age 58+	−0.034
	(0.069)
Female	−0.185*
	(0.044)
Asian	0.002
	(0.085)
Black	0.484*
	(0.126)
Mixed	−0.177
	(0.186)
Other ethnicity	0.224
	(0.241)
Observations	1,694
R^2	0.127
Adjusted R^2	0.121
Residual Std. Error	0.893 (df = 1681)
F Statistic	20.460* (df = 12; 1681)
Note:	*$p < 0.05$

Table 18: Full model summary, effect of optimism and hope on quality of life pre-election prospective evaluation.

	Dependent variable:
	Pre-election: quality of life (will get much/slightly better/worse)
Intercept	2.036*
	(0.108)
Optimism	0.987*
	(0.126)
Hope	0.204
	(0.161)
Prefer Labour	−0.591*
	(0.048)
Age 28-37	−0.121
	(0.071)
Age 38-47	−0.023
	(0.070)
Age 48-57	−0.078
	(0.073)
Age 58+	−0.057
	(0.066)
Female	−0.080
	(0.041)
Asian	−0.024
	(0.081)
Black	0.503*
	(0.120)
Mixed	−0.261
	(0.177)
Other ethnicity	−0.271
	(0.229)
Observations	1,693
R^2	0.175
Adjusted R^2	0.169
Residual Std. Error	0.851 (df = 1680)
F Statistic	29.745* (df = 12; 1680)
Note:	*$p < 0.05$

Table 19: Full model summary, effect of optimism and hope on carbon emission post-election prospective evaluation.

	Dependent variable:
	Post-election: carbon emissions (will get much/slightly better/worse)
Intercept	2.584*
	(0.113)
Optimism	0.378*
	(0.131)
Hope	0.395*
	(0.168)
Prefer Labour	−0.083
	(0.051)
Age 28-37	−0.004
	(0.074)
Age 38-47	0.068
	(0.073)
Age 48-57	0.133
	(0.076)
Age 58+	0.091
	(0.069)
Female	−0.209*
	(0.043)
Asian	−0.023
	(0.085)
Black	0.107
	(0.126)
Mixed	−0.012
	(0.185)
Other ethnicity	0.109
	(0.240)
Observations	1,694
R^2	0.044
Adjusted R^2	0.037
Residual Std. Error	0.890 (df = 1681)
F Statistic	6.407* (df = 12; 1681)
Note:	$^*p < 0.05$

Table 20: Full model summary, effect of optimism and hope on climate crisis post-election prospective evaluation.

	Dependent variable:
	Post-election: climate crisis (will get much/slightly better/worse)
Intercept	2.069*
	(0.114)
Optimism	0.519*
	(0.133)
Hope	0.450*
	(0.171)
Prefer Labour	−0.273*
	(0.052)
Age 28-37	0.065
	(0.076)
Age 38-47	0.137
	(0.074)
Age 48-57	0.137
	(0.078)
Age 58+	0.090
	(0.070)
Female	−0.038
	(0.044)
Asian	0.002
	(0.086)
Black	0.253*
	(0.128)
Mixed	−0.238
	(0.188)
Other ethnicity	0.489*
	(0.244)
Observations	1,694
R^2	0.066
Adjusted R^2	0.059
Residual Std. Error	0.905 (df = 1681)
F Statistic	9.855* (df = 12; 1681)
Note:	*$p < 0.05$

Table 21: Full model summary, effect of optimism and hope on economy post-election prospective evaluation.

	Dependent variable:
	Post-election: economy (will get much/slightly better/worse)
Intercept	2.050*
	(0.131)
Optimism	0.835*
	(0.152)
Hope	0.384*
	(0.195)
Prefer Labour	0.029
	(0.059)
Age 28-37	0.105
	(0.086)
Age 38-47	0.307*
	(0.084)
Age 48-57	0.328*
	(0.089)
Age 58+	0.289*
	(0.080)
Female	−0.180*
	(0.050)
Asian	−0.019
	(0.098)
Black	0.249
	(0.146)
Mixed	−0.286
	(0.215)
Other ethnicity	−0.148
	(0.278)
Observations	1,694
R^2	0.073
Adjusted R^2	0.066
Residual Std. Error	1.034 (df = 1681)
F Statistic	11.048* (df = 12; 1681)
Note:	$^*p < 0.05$

Table 22: Full model summary, effect of optimism and hope on NHS post-election prospective evaluation.

	Dependent variable:
	Post-election: NHS (will get much/slightly better/worse)
Intercept	2.037*
	(0.132)
Optimism	0.936*
	(0.154)
Hope	0.028
	(0.198)
Prefer Labour	0.247*
	(0.060)
Age 28-37	−0.005
	(0.087)
Age 38-47	0.149
	(0.085)
Age 48-57	0.172
	(0.090)
Age 58+	0.243*
	(0.081)
Female	−0.200*
	(0.051)
Asian	0.016
	(0.099)
Black	0.228
	(0.148)
Mixed	−0.162
	(0.217)
Other ethnicity	0.048
	(0.282)
Observations	1,694
R^2	0.062
Adjusted R^2	0.056
Residual Std. Error	1.046 (df = 1681)
F Statistic	9.311* (df = 12; 1681)
Note:	$^{*}p < 0.05$

Table 23: Full model summary, effect of optimism and hope on quality of life post-election prospective evaluation.

	Dependent variable:
	Post-election: quality of life (will get much/slightly better/worse)
Intercept	1.992*
	(0.122)
Optimism	0.873*
	(0.143)
Hope	0.381*
	(0.183)
Prefer Labour	0.087
	(0.055)
Age 28-37	−0.0001
	(0.081)
Age 38-47	0.155*
	(0.079)
Age 48-57	0.185*
	(0.083)
Age 58+	0.141
	(0.075)
Female	−0.089
	(0.047)
Asian	0.009
	(0.092)
Black	0.139
	(0.137)
Mixed	−0.190
	(0.201)
Other ethnicity	−0.160
	(0.261)
Observations	1,694
R^2	0.067
Adjusted R^2	0.060
Residual Std. Error	0.969 (df = 1681)
F Statistic	10.012* (df = 12; 1681)
Note:	$^*p < 0.05$

3.3 Subscale effects

Figures 3 and 5 display the effects of hope and optimism subscales on each of our prospective evaluations measures. The left half of Figure 3 displays the effects of optimism on the perceived likelihood of each potential future outcome, using only the positively valenced items from the Life Orientation Test (optimism only), and using only the negatively valenced items (pessimism only), controlling in both cases for the full hope scale. The right half of Figure 3 displays the effects of the agency and pathway subscales of the Adult Hope Scale, controlling in both cases for the full optimism scale. Figure 4 displays the effects of the optimism and pessimism subscales Life Orientation Test, dervied from the same model in which both are included as predictors. Figure 5 displays this same information for the items asking respondents whether a range of outcomes will get better or worse in the future. On the whole, these subscale analyses produce results that are very consistent with our main analyses using the full optimism and hope scales, providing further support for our findings. However, Figure 5 suggests that the negative overall effect of optimism on expectations for negatively valenced outcomes may be driven especially by pessimism, rather than just low rates of optimism.

3.4 Bayesian models

In the main text, when modelling our get better/get worse prospective evaluations outcomes, we followed the common practice of using OLS models for likert outcome data. However, this approach often gives rise to false positives (Type I errors), false negatives (Type II error), and even estimates with incorrect signs (Type S errors) (Liddell and Kruschke 2018) – problems which may not be solved by the common alternative of using frequentist ordered logit methods (Bürkner and Vuorre 2019). Here, we therefore

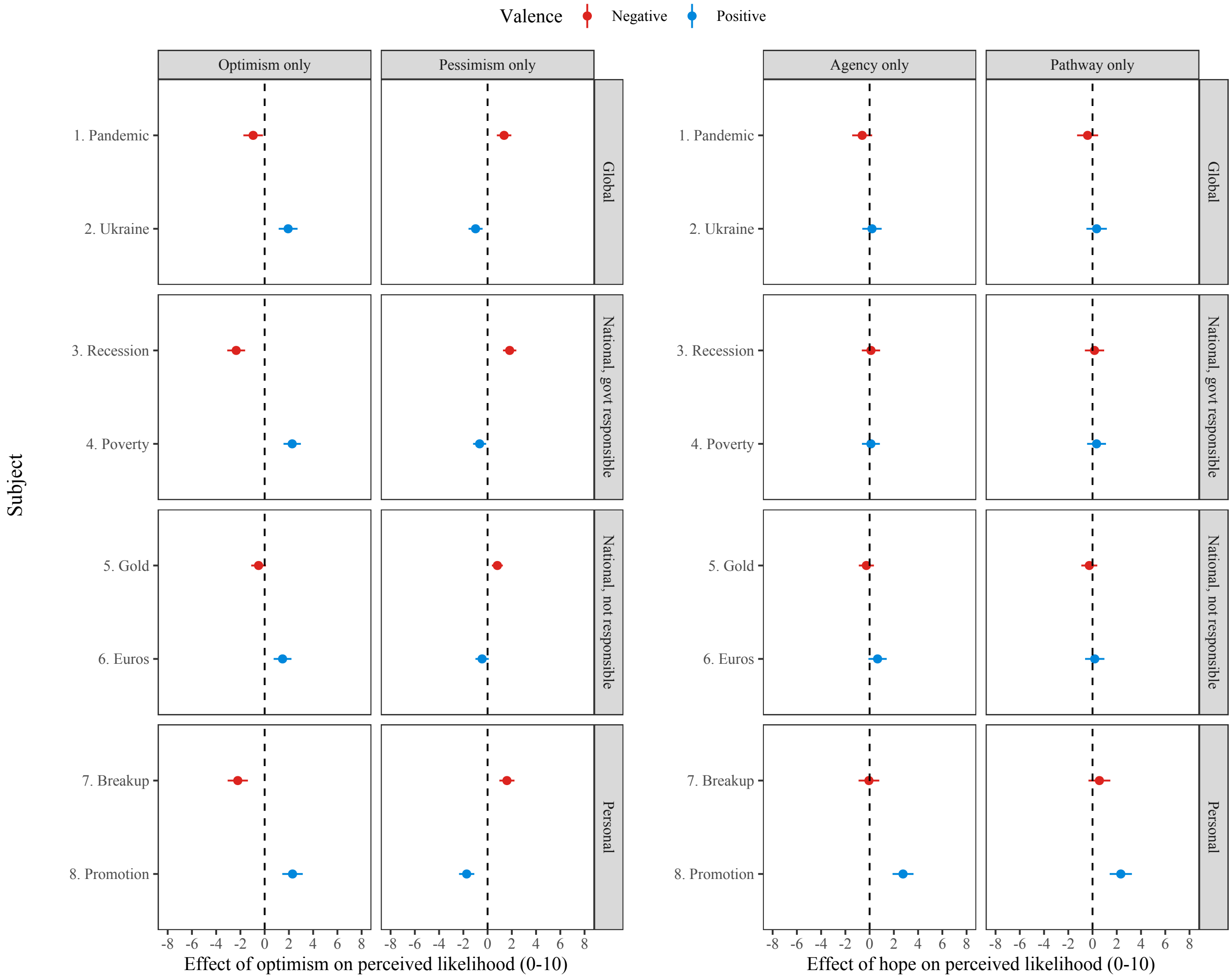

Figure 3: Effects (and 95% CI) of optimism/hope subscales on perceived likelihoods of future outcomes.

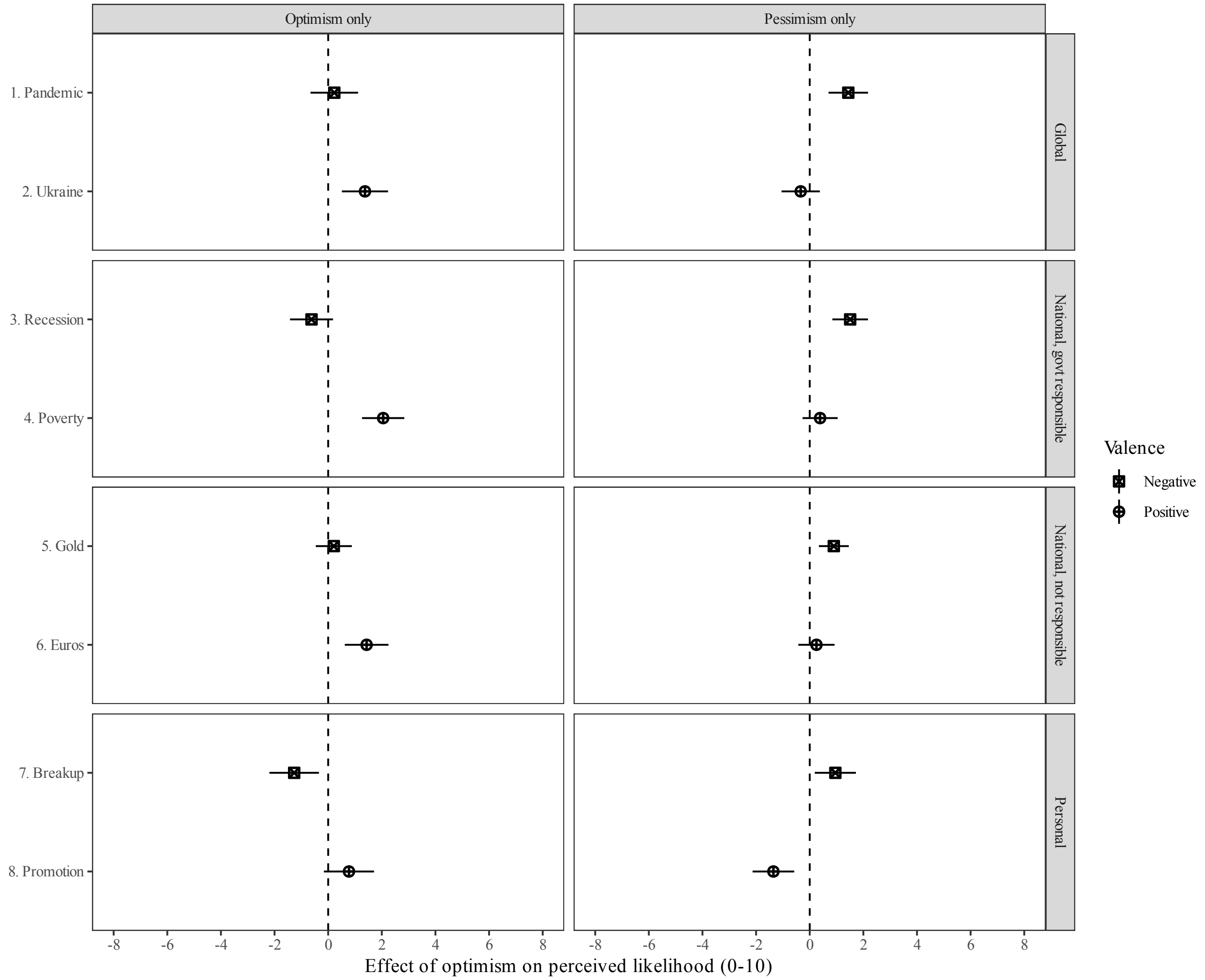

Figure 4: Effects (and 95% CI) of optimism and pessimism subscales on perceived likelihoods of future outcomes, derived from model including both.

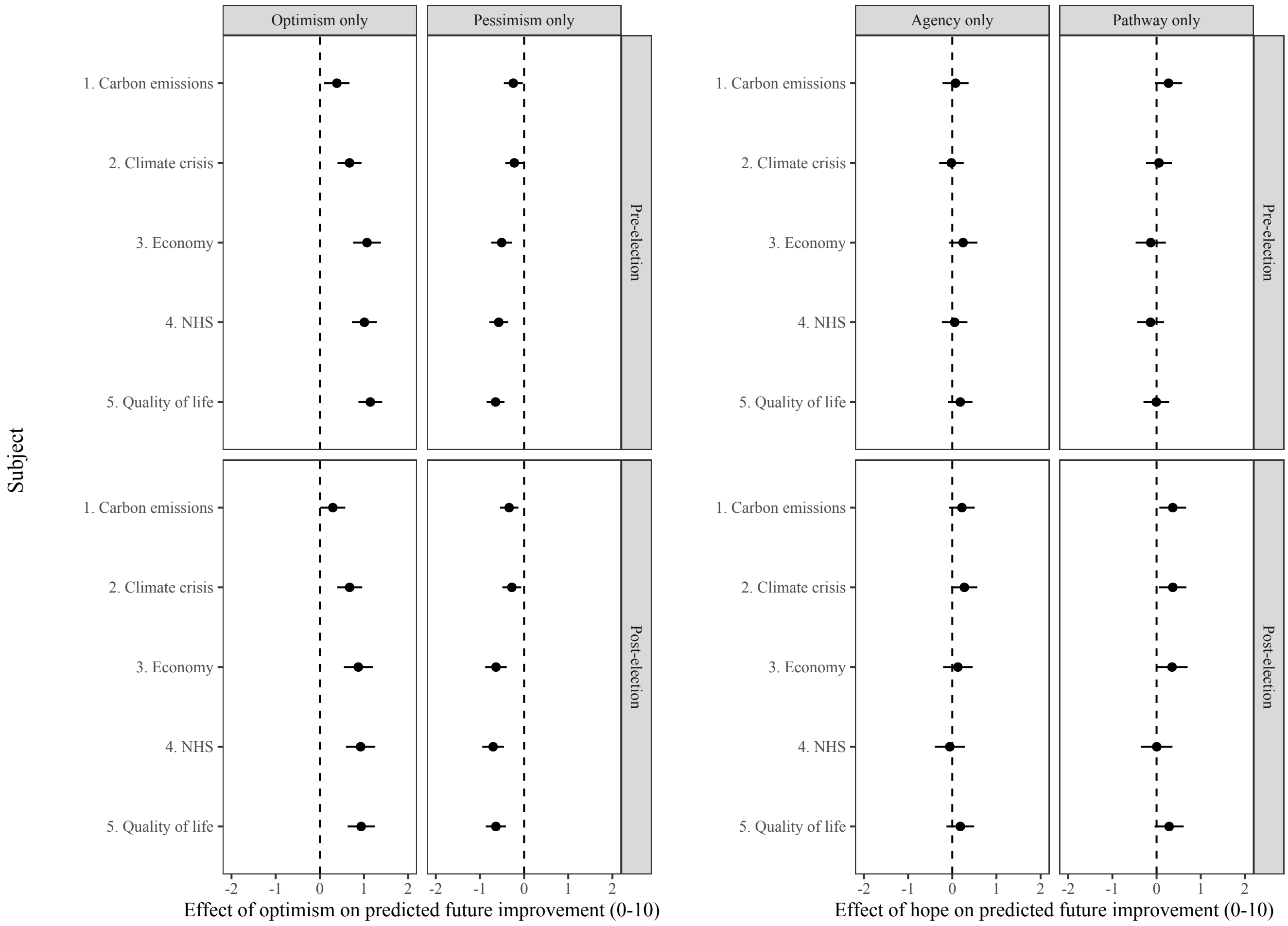

Figure 5: Effects (and 95% CI) of optimism/hope subscales on predicted future improvement of societal outcomes.

follow Liddell and Kruschke's (2018), Bürkner and Vuorre's (2019), McElreath's (2020) advice to use Bayesian ordinal regression models specifically tailored for modelling ordinal outcome data (see Bürkner and Vuorre 2019 for a full exposition of the method).

For each of the two groups of get better/get worse outcomes – pre-election and post-election – we partially pool across the items, by pooling the data and including a varying intercept for each item. Tables 24 and 25 present summaries of the models including the effect of each variable on the latent mean of the outcome, discrimination parameters, threshold parameters, and group-level standard deviations of intercepts. As these are Bayesian models, the output is a full posterior distribution of each coefficient. Tables 24 and 25 summarise these distributions by their median, error, and the upper and lower bounds of their 95% Bayesian credible interval. We use Bailey's (2021) recommended conservative priors for these models.

The results show that, consistent with the findings from our OLS models, optimism clearly drives up beliefs that political outcomes will improve rather than worsen in the coming years, both before and after the election – holding constant party preferences and demographic characteristics. Hope has no equivalent effect in the pre-election period, but does appear to produce slightly more positive prospective evaluations for the post-election period, consistent with the finding reported in the main text that more hopeful people are more likely to believe the climate crisis and the UK's carbon emissions will improve after the election. Interestingly, these results also show that pre-election prospective evaluations are strongly partisan – those who prefer Labour over the Conservatives are substantially less likely to say things will improve in this period, while the Conservatives are still in government. However, for post-election prospective evaluations, this difference is wiped out, likely because Labour supporters believe their party will be in power after the election.

Table 24: Summary of Bayesian ordinal regression model predicting pre-election prospective evaluations.

Variable	Median	Error	2.5%	97.5%	Variable	Median	Error	2.5%	97.5%
**Mean**					**Discrimination**				
Optimism	0.87	0.13	0.59	1.10	Optimism	0.09	0.06	-0.03	0.22
Hope	0.11	0.09	-0.04	0.29	Hope	-0.35	0.08	-0.50	-0.20
Prefer Labour	-0.58	0.09	-0.72	-0.39	Prefer Labour	-0.03	0.02	-0.08	0.02
Age 28-37	0.00	0.04	-0.08	0.08	Age 28-37	0.09	0.04	0.02	0.16
Age 38-47	0.08	0.04	0.01	0.17	Age 38-47	0.05	0.03	-0.02	0.11
Age 48-57	0.05	0.04	-0.02	0.14	Age 48-57	0.14	0.04	0.07	0.21
Age 58+	0.08	0.04	0.01	0.16	Age 58+	0.09	0.03	0.03	0.15
Gender	-0.18	0.03	-0.25	-0.11	Gender	-0.05	0.02	-0.09	-0.01
Asian	-0.03	0.04	-0.11	0.05	Asian	0.14	0.04	0.06	0.22
Black	0.38	0.09	0.22	0.57	Black	-0.23	0.06	-0.34	-0.13
Mixed	-0.15	0.10	-0.35	0.03	Mixed	0.03	0.09	-0.15	0.19
Other ethnicity	0.11	0.11	-0.12	0.34	Other ethnicity	0.02	0.10	-0.19	0.22

Variable	Median	Error	2.5%	97.5%
**Thresholds**				
Threshold 1	-0.76	0.25	-1.36	-0.37
Threshold 2	0.36	0.21	-0.12	0.70
Threshold 3	1.17	0.25	0.64	1.62
Threshold 4	2.68	0.41	1.77	3.39
**Random effects**				
sd(Outcome item)	0.35	0.15	0.17	0.97
sd(Outcome item disc)	0.24	0.16	0.08	1.00
N				1694.00

Table 25: Summary of Bayesian ordinal regression model predicting post-election prospective evaluations.

Variable	Median	Error	2.5%	97.5%	Variable	Median	Error	2.5%	97.5%
**Mean**					**Discrimination**				
Optimism	0.86	0.11	0.63	1.07	Optimism	0.08	0.06	-0.04	0.19
Hope	0.41	0.10	0.22	0.61	Hope	-0.31	0.07	-0.44	-0.17
Prefer Labour	-0.02	0.03	-0.08	0.03	Prefer Labour	-0.08	0.02	-0.13	-0.04
Age 28-37	0.04	0.04	-0.05	0.12	Age 28-37	0.03	0.03	-0.03	0.10
Age 38-47	0.18	0.05	0.09	0.27	Age 38-47	0.01	0.03	-0.06	0.07
Age 48-57	0.20	0.05	0.11	0.30	Age 48-57	0.05	0.03	-0.02	0.11
Age 58+	0.18	0.04	0.10	0.27	Age 58+	0.05	0.03	-0.01	0.11
Gender	-0.16	0.03	-0.22	-0.10	Gender	0.00	0.02	-0.04	0.03
Asian	-0.02	0.04	-0.11	0.07	Asian	0.13	0.04	0.06	0.21
Black	0.23	0.08	0.08	0.39	Black	-0.12	0.05	-0.22	-0.02
Mixed	-0.18	0.10	-0.38	0.02	Mixed	-0.01	0.08	-0.16	0.15
Other ethnicity	0.06	0.11	-0.15	0.27	Other ethnicity	0.20	0.10	-0.01	0.39

Variable	Median	Error	2.5%	97.5%
**Thresholds**				
Threshold 1	-0.62	0.16	-0.97	-0.34
Threshold 2	0.43	0.14	0.13	0.71
Threshold 3	1.41	0.20	0.99	1.78
Threshold 4	3.29	0.37	2.45	3.91
**Random effects**				
sd(Outcome item)	0.35	0.15	0.17	0.97
sd(Outcome item disc)	0.24	0.16	0.08	1.00
N				1694.00

4 Electoral expectations

4.1 Distributions of expectations

Figure 6 displays the distribution of responses to our focal electoral expectations items asking respondents to predict how likely it was that the Labour and Conservative Party would win a majority at the next election. The distributions show that for supporters of both parties, expectations for the Labour Party were fairly high: the perceived likelihood of the Labour Party forming a majority is clustered mostly around 4-8/10 on the scale. However, Labour supporters' expectations are especially high, with a modal perceived probability of 8/10. for the Conservative Party meanwhile, Labour supporters' expectations are clustered towards the lower end of the scale, while Conservative supporters' expectations are approximately normally distributed with a mode of 5/10.

4.2 Liberal Democrat expectations

Figure 7 displays the distribution of responses to the electoral expectation item asking respondents to predict how likely it was that the Liberal Democrats would win a majority at the next election. Clearly, among both groups, expectations that the Lib Dems could win the election were very low, with a modal response of 0/10. There are minimal signs of differences between Labour and Conservative supporters in their responses to this item.

Figure 8 displays the effects of our treatments on Liberal Democrat expectations. The results reveal that both the poll ($\beta =$ -0.27, 95% CI: -0.50, -0.05) and the poll combined with expert doubt (about the Labour lead over the Conservatives) statistically significantly lowered expectations that the Liberal Democrats

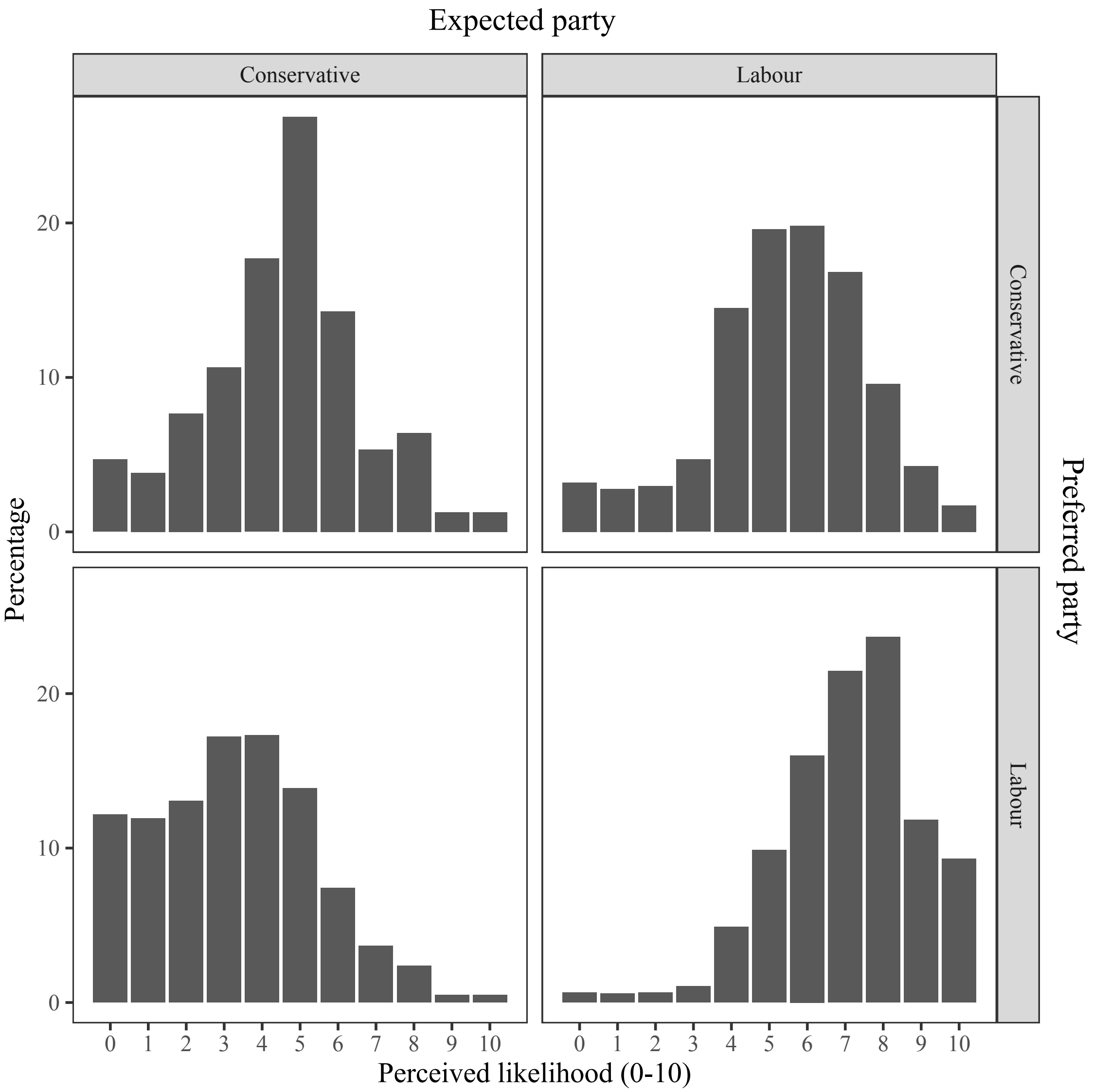

Figure 6: Distributions of responses to main electoral expectations items, among supporters of Conservative and Labour.

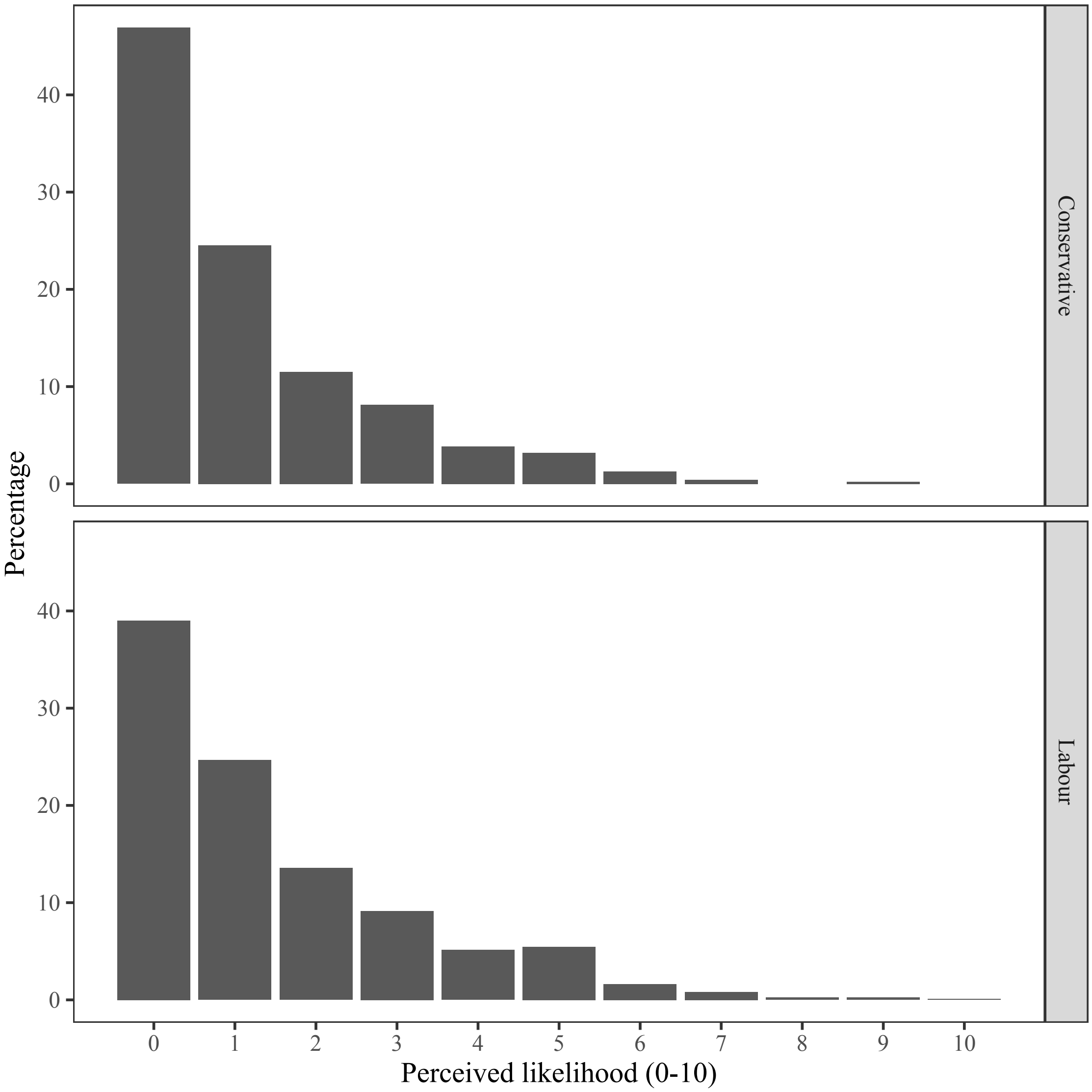

Figure 7: Distributions of responses to Liberal Democrat electoral expectations, among supporters of Conservative and Labour.

could win the next election (β = -0.27, 95% CI: -0.49, -0.05). The estimate for the effect of the poll reinforced by an expert statement (reaffiriming Labour's lead over the Conservatives) is also negative and of a similar size but not statistically significant (β = -0.20, 95% CI: -0.42, 0.02). On the whole then, exposure to polling information likely lowered perceptions of the Liberal Democrats' chances – unsurprisingly, given that the party had a share of only 10% in the poll.

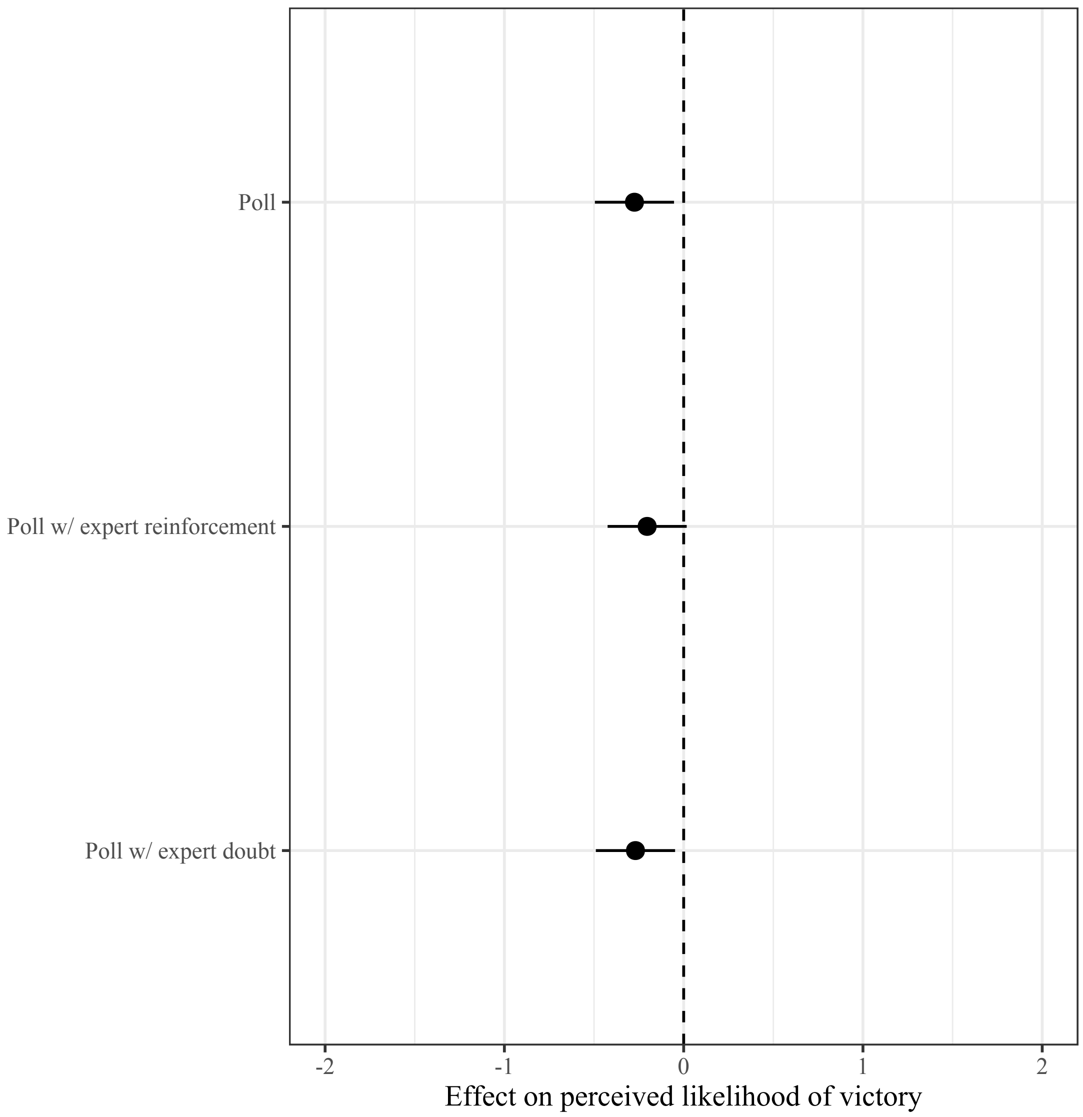

Figure 8: Effects (and 95% CI) of treatment conditions on electoral expectations for Liberal Democrats, versus pure control condition, controlling for age, gender, ethnicity, and preferred winner.

4.3 Full sample interactions

In the main text, we present the interactive effect of optimism/hope and party preference on electoral expectations. As those expectations are measured post-treatment, we focus only on the control group to have an unmanipulated measure of expectations. In Figure 9 below we conduct the same analysis, but with the full sample, controlling statistically for treatment status. We uncover the same general patterns as reported in the main text, but with more precision, owing to the much larger sample size.

4.4 Model summaries

Tables 26-29 provide full tabulated summaries of the electoral expectations models reported in the main text. Table 26 reports the full results of the model estimating the interaction effects between party preferences and hope and optimism on the perceived difference in the parties' chances amongst our control group. Table 28 presents the full results of the models estimating the average effects of our treatments on expectations for each party. Table 27 reports the results of the model estimating the interaction effects between treatment condition and party preferences. Table 29 reports the results of the model estimating the interaction effects between treatment condition and optimism/hope.

4.5 Subscale effects

Figure 10 displays the effects of hope and optimism subscales on electoral expectations. The left half of Figure 10 displays the effects of optimism on the difference in the perceived likelihood of a Labour majority over a Conservative majority, using only the positively valenced items from the Life Orientation Test (optimism only), and using only the negatively valenced items (pessimism only), controlling in both

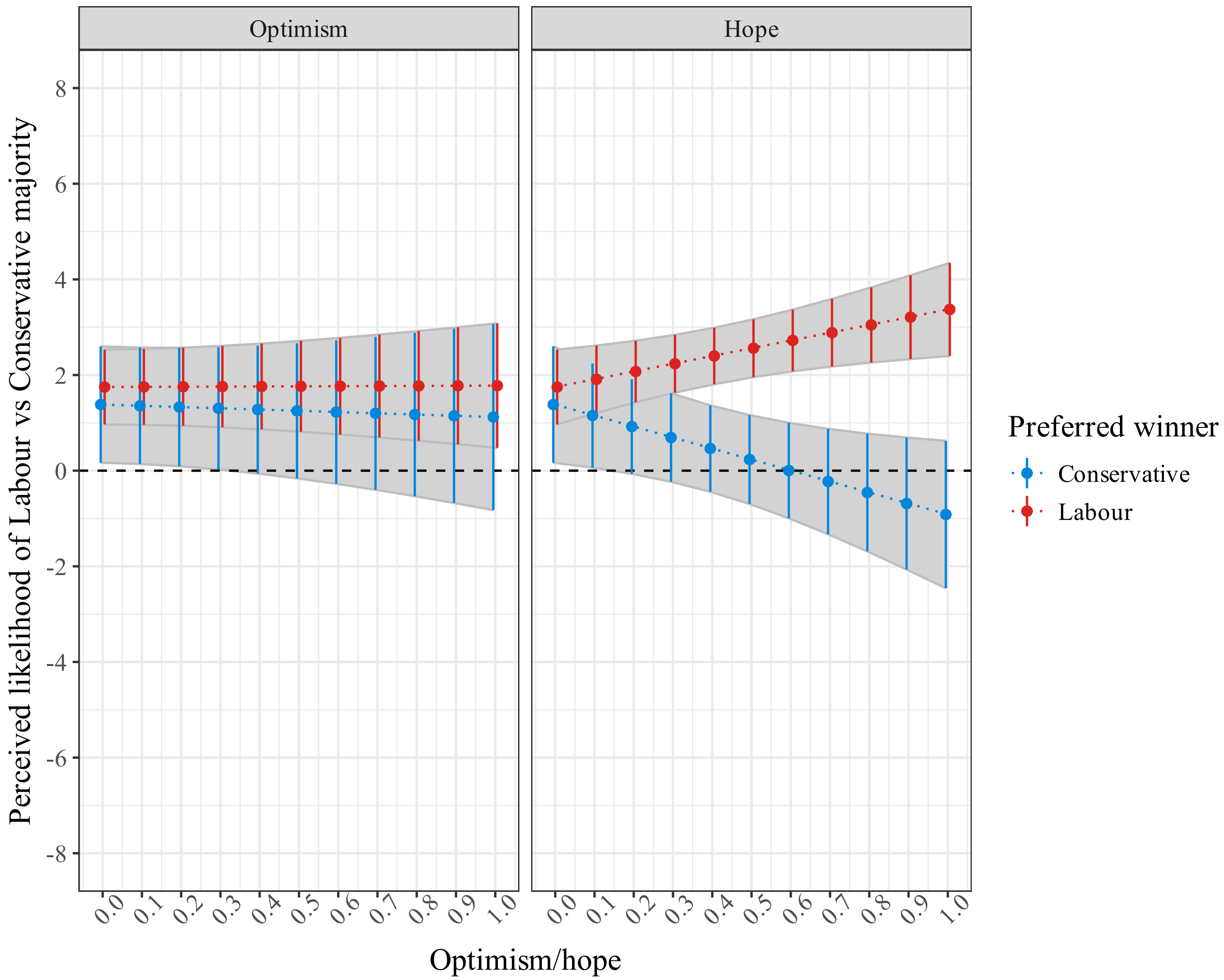

Figure 9: Predicted difference (and 95% CI) between perceived likelihood of Labour majority and perceived likelihood of Conservative majority, between Labour and Conservative supporters, by levels optimism and hope. Predictions taken from one model including interaction terms for both optimism and hope, with controls for treatment condition, age, gender, and ethnicity.

Table 26: Full model summary, effect of optimism and hope on electoral expectations and interaction with party preference.

	Dependent variable:
	Difference in likelihood of majority (Labour - Conservative)
Intercept	1.382*
	(0.620)
Prefer Labour	0.366
	(0.661)
Optimism	−0.261
	(0.813)
Hope	−2.299*
	(1.052)
Poll treatment	0.785*
	(0.204)
Poll Ford treatment	−0.202
	(0.204)
Poll Jennings treatment	1.217*
	(0.204)
Age 28-37	0.515*
	(0.247)
Age 38-47	1.418*
	(0.242)
Age 48-57	1.791*
	(0.254)
Age 58+	1.191*
	(0.228)
Female	−0.726*
	(0.144)
Asian	−0.045
	(0.281)
Black	−0.074
	(0.418)
Mixed	−0.585
	(0.615)
Other ethnicity	0.085
	(0.796)
Prefer Labour:Optimism interaction	0.290
	(0.954)
Prefer Labour:Hope interaction	3.922*
	(1.237)
Observations	1,694
R^2	0.232
Adjusted R^2	0.224
Residual Std. Error	2.956 (df = 1676)
F Statistic	29.699* (df = 17; 1676)
Note:	$^*p < 0.05$

Table 27: Full model summaries, effect of treatment conditions on expectations for each party.

	Dependent variable:	
	Labour expectations	Conservative expectations
	(1)	(2)
Intercept	5.054*	5.372*
	(0.171)	(0.183)
Poll treatment	0.358*	−0.449*
	(0.131)	(0.141)
Poll Ford treatment	0.054	0.248
	(0.131)	(0.141)
Poll Jennings treatment	0.422*	−0.841*
	(0.131)	(0.141)
Prefer Labour	1.666*	−1.445*
	(0.108)	(0.116)
Age 28-37	0.075	−0.425*
	(0.158)	(0.170)
Age 38-47	0.530*	−0.899*
	(0.155)	(0.167)
Age 48-57	0.477*	−1.304*
	(0.162)	(0.174)
Age 58+	0.359*	−0.809*
	(0.144)	(0.155)
Female	−0.274*	0.436*
	(0.093)	(0.099)
Asian	0.132	0.201
	(0.181)	(0.194)
Black	0.443	0.431
	(0.268)	(0.288)
Mixed	−0.272	0.261
	(0.394)	(0.423)
Other ethnicity	0.103	−0.008
	(0.512)	(0.550)
Observations	1,694	1,694
R^2	0.147	0.149
Adjusted R^2	0.140	0.142
Residual Std. Error (df = 1680)	1.903	2.045
F Statistic (df = 13; 1680)	22.280*	22.545*
Note:		$^*p < 0.05$

Table 28: Full model summaries, effect of treatment conditions on perceived difference in parties' chances with interaction by party preference.

	Dependent variable:
	Difference in likelihood of majority (Labour - Conservative)
Intercept	−0.114
	(0.323)
Poll treatment	0.460
	(0.376)
Poll Ford treatment	−0.489
	(0.394)
Poll Jennings treatment	1.031*
	(0.384)
Prefer Labour	2.805*
	(0.318)
Age 28-37	0.510*
	(0.248)
Age 38-47	1.447*
	(0.243)
Age 48-57	1.784*
	(0.253)
Age 58+	1.175*
	(0.225)
Female	−0.709*
	(0.145)
Asian	−0.071
	(0.282)
Black	0.015
	(0.418)
Mixed	−0.552
	(0.616)
Other ethnicity	0.125
	(0.801)
Prefer Labour:Poll interaction	0.494
	(0.448)
Prefer Labour:Poll Ford interaction	0.415
	(0.461)
Prefer Labour:Poll Jennings interaction	0.332
	(0.454)
Observations	1,694
R^2	0.223
Adjusted R^2	0.216
Residual Std. Error	2.971 (df = 1677)
F Statistic	30.072* (df = 16; 1677)
Note:	*$p < 0.05$

Table 29: Full model summaries, effect of interaction between treatment condition and optimism/hope on perceptions of preferred party's advantage over opponent.

	Dependent variable:	
	Difference in likelihood of majority (preferred party - other party)	
	(1)	(2)
Intercept	−2.751*	−4.887*
	(0.460)	(0.672)
Poll treatment	0.517	2.187*
	(0.567)	(0.851)
Poll w/ in-boost treatment	1.042	2.472*
	(0.587)	(0.887)
Poll w/out-boost treatment	0.049	1.818*
	(0.560)	(0.822)
Optimism	1.069	
	(0.674)	
Hope		4.054*
		(0.928)
Prefer Labour	4.971*	5.000*
	(0.172)	(0.171)
Age 28-37	0.666*	0.673*
	(0.252)	(0.250)
Age 38-47	1.153*	1.167*
	(0.247)	(0.245)
Age 48-57	1.178*	1.244*
	(0.258)	(0.256)
Age 58+	1.168*	1.232*
	(0.232)	(0.227)
Female	−0.192	−0.163
	(0.147)	(0.146)
Asian	−0.054	−0.051
	(0.287)	(0.285)
Black	0.265	0.242
	(0.427)	(0.423)
Mixed	−0.552	−0.578
	(0.627)	(0.622)
Other ethnicity	−0.199	−0.260
	(0.814)	(0.808)
Optimism:Poll interaction	−0.076	
	(0.939)	
Optimism:Poll w/ in-boost interaction	0.067	
	(0.971)	
Optimism:Poll w/ out-boost interaction	−0.728	
	(0.933)	
Hope:Poll interaction		−2.591*
		(1.262)
Hope:Poll w/ in-boost interaction		−2.128
		(1.305)
Hope:Poll w/ out-boost interaction		−3.300*
		(1.223)
Observations	1,694	1,694
R^2	0.361	0.368
Adjusted R^2	0.354	0.362
Residual Std. Error (df = 1676)	3.018	3.001
F Statistic (df = 17; 1676)	55.624*	57.432*
Note:		$^*p < 0.05$

cases for the full hope scale. The right half of Figure 10 displays the effects of the agency and pathway subscales of the Adult Hope Scale, controlling in both cases for the full optimism scale. On the whole, these subscale analyses produce results that are very consistent with our main analyses using the full optimism and hope scales, providing further support for our findings.

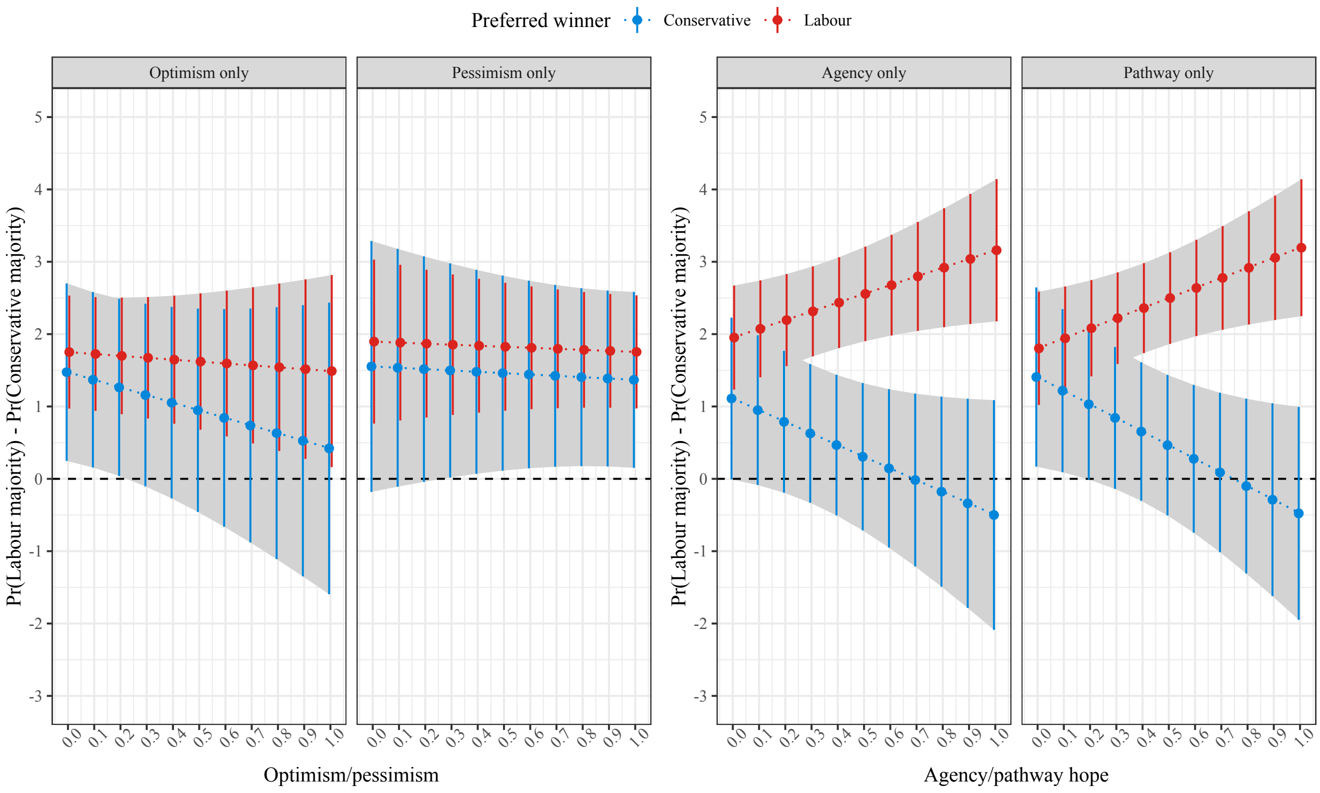

Figure 10: Effects (and 95% CI) of optimism/hope subscales on perceived difference in parties' chances of forming a majority.

4.6 Majority/coalition items

As a secondary measure of electoral expectations, we measured respondents' perceived most likely election outcomes, between a Labour majority government, Labour-led coalition government, Conservative majority government, or Conservative-led coalition government.

Figure 11 displays the distributions of these perceived most likely election outcomes among Conservative

and Labour supporters. Labour supporters (62.5%) are much more likely than Conservative supporters (32.8%) to predict a Labour majority, although this is the modal prediction among both groups. Conservatives (22.4%) are much more likely to predict a Conservative-led coalition than Labour supporters (4.1%), for whom it is the least likely outcome. These patterns provide further evidence of partisan bias in electoral expectations.

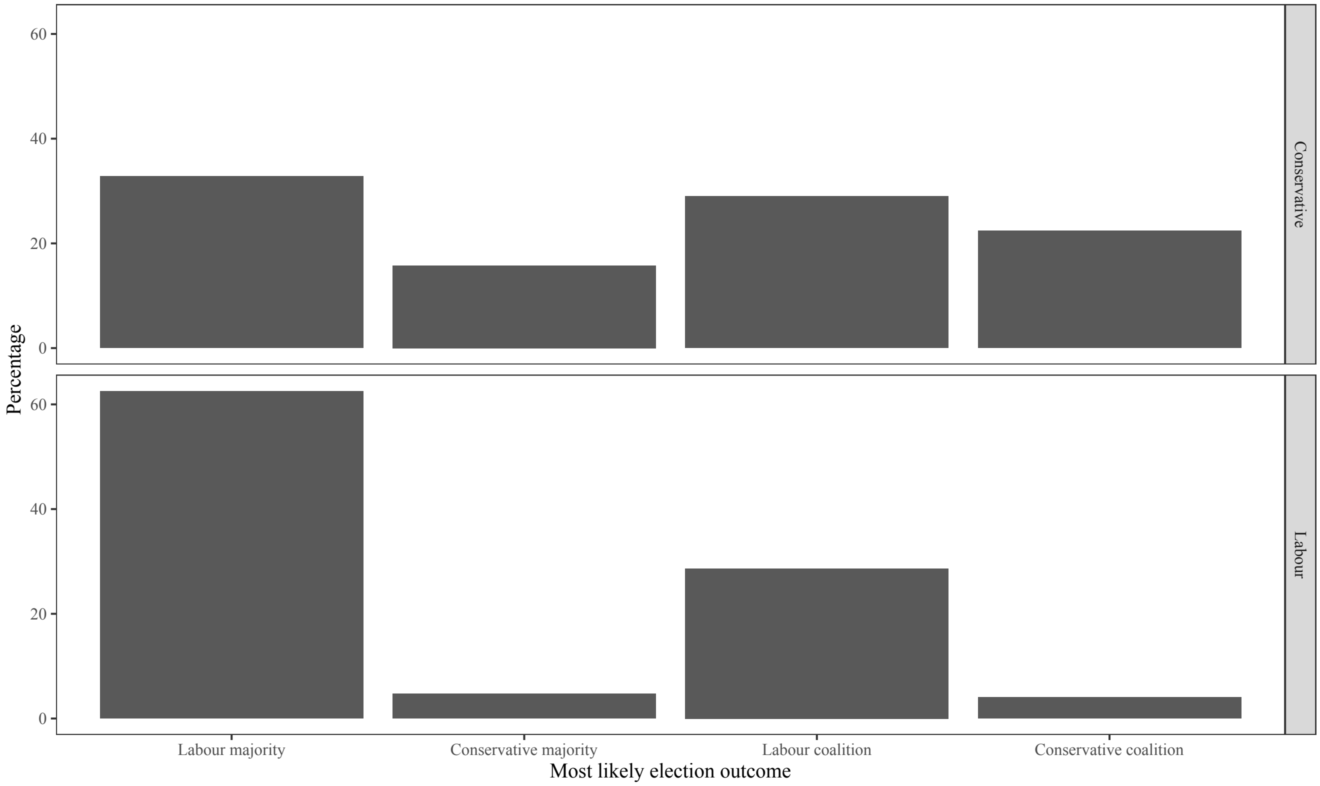

Figure 11: Distributions of perceived most likely election outcome, by party preference.

To assess the robustness of the effects of hope and optimism on partisan electoral expectations reported in the main text, we reconduct these analyses with this alternative measure of electoral expectations as our outcome variable. For the purpose of these analyses, we recode this variable such that any prediction of a Conservative government (majority *or* coalition) takes the value 0, and any Labour government prediction takes the value 1.

Figures 12 and 13 display the interactive effect of optimism/hope and electoral preferences on the probability of choosing a Labour government over a Conservative government as the most likely outcome, in the full sample and the control group only, respectively. The results confirm those reported in the main text (in Figure 4). Optimism does not moderate the effect of electoral preference on expectations, but hope does. Indeed, there is no meaningful difference in the predicted expectations of opposing partisans low in hope, and large, significant differences in opposing partisans high in hope.

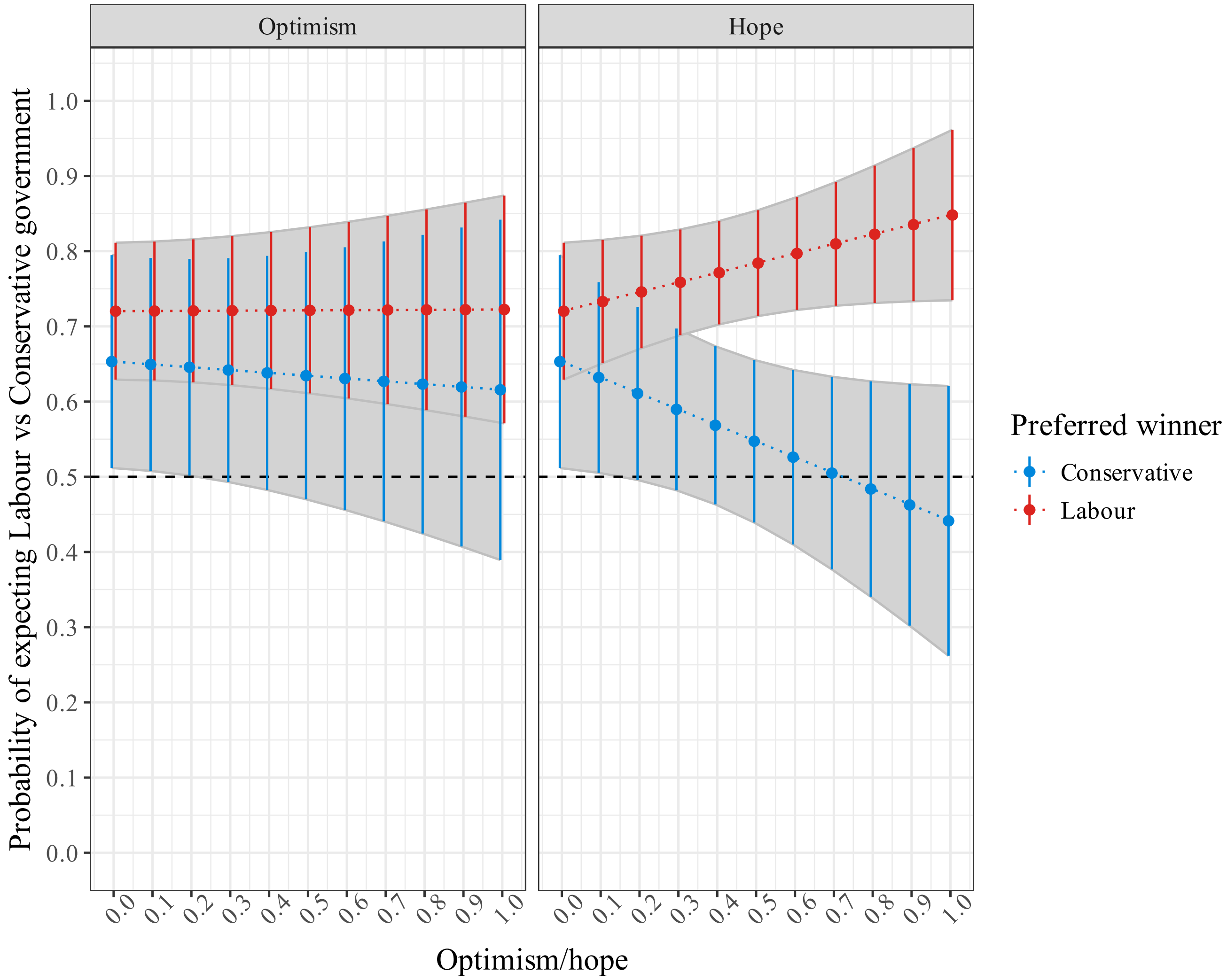

Figure 12: Predicted probability (and 95% CI) of choosing a Labour rather than Conservative government as most likely outcome, between Labour and Conservative supporters, by levels optimism and hope, in full sample, controlling for treatment.

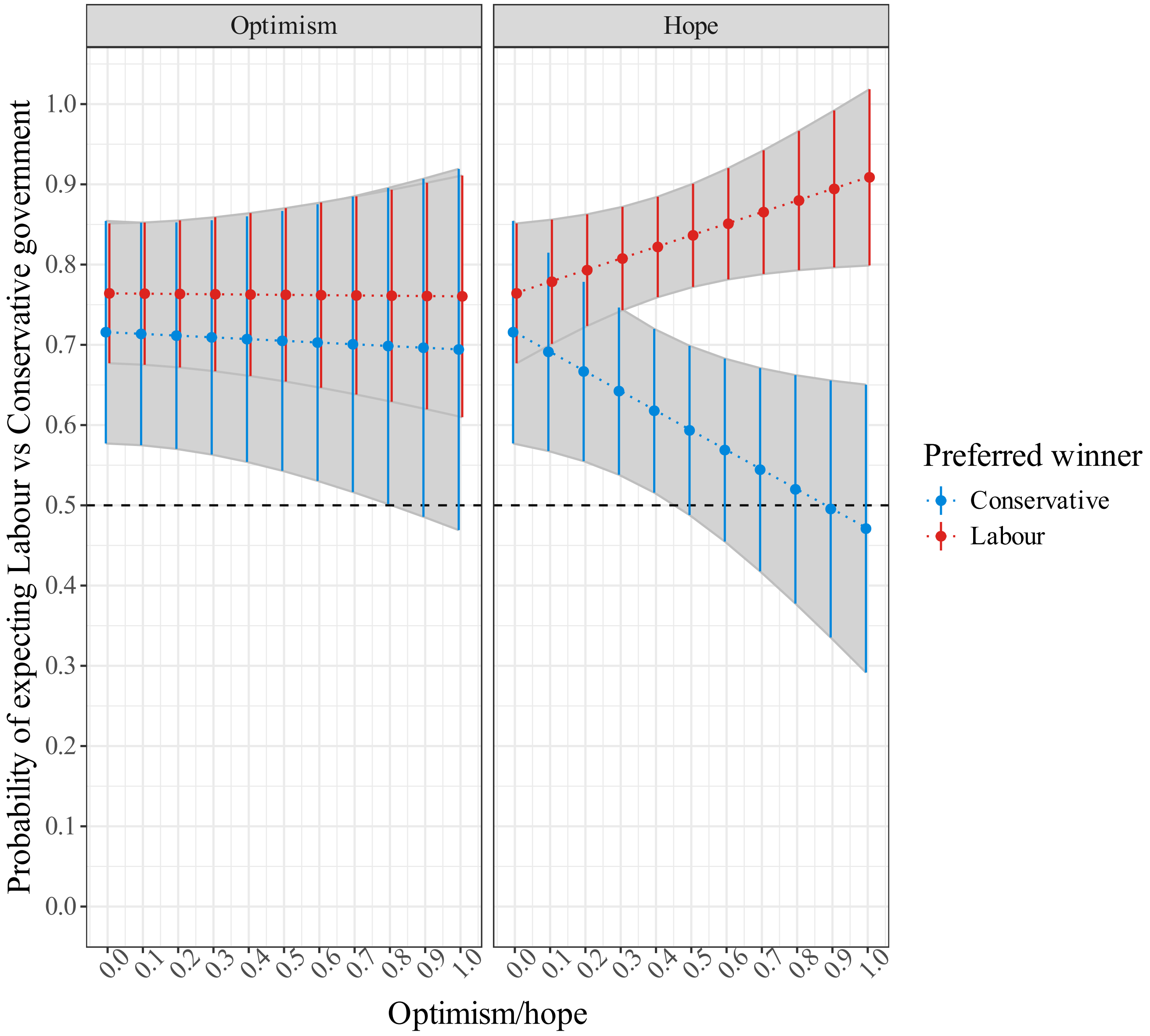

Figure 13: Predicted probability (and 95% CI) of choosing a Labour rather than Conservative government as most likely outcome, between Labour and Conservative supporters, by levels optimism and hope, in control group only.

We also verify that treatment significantly affected this measure of expectations in line with its effects reported in the main text. Figure 14 shows that, consistent with our primary measure of electoral expectations, both the Poll and Poll with expert reinforcement treatments significantly raise the probability of selecting a Labour government as the most likely outcome, but under the Poll with expert doubt treatment this effect is cancelled out.

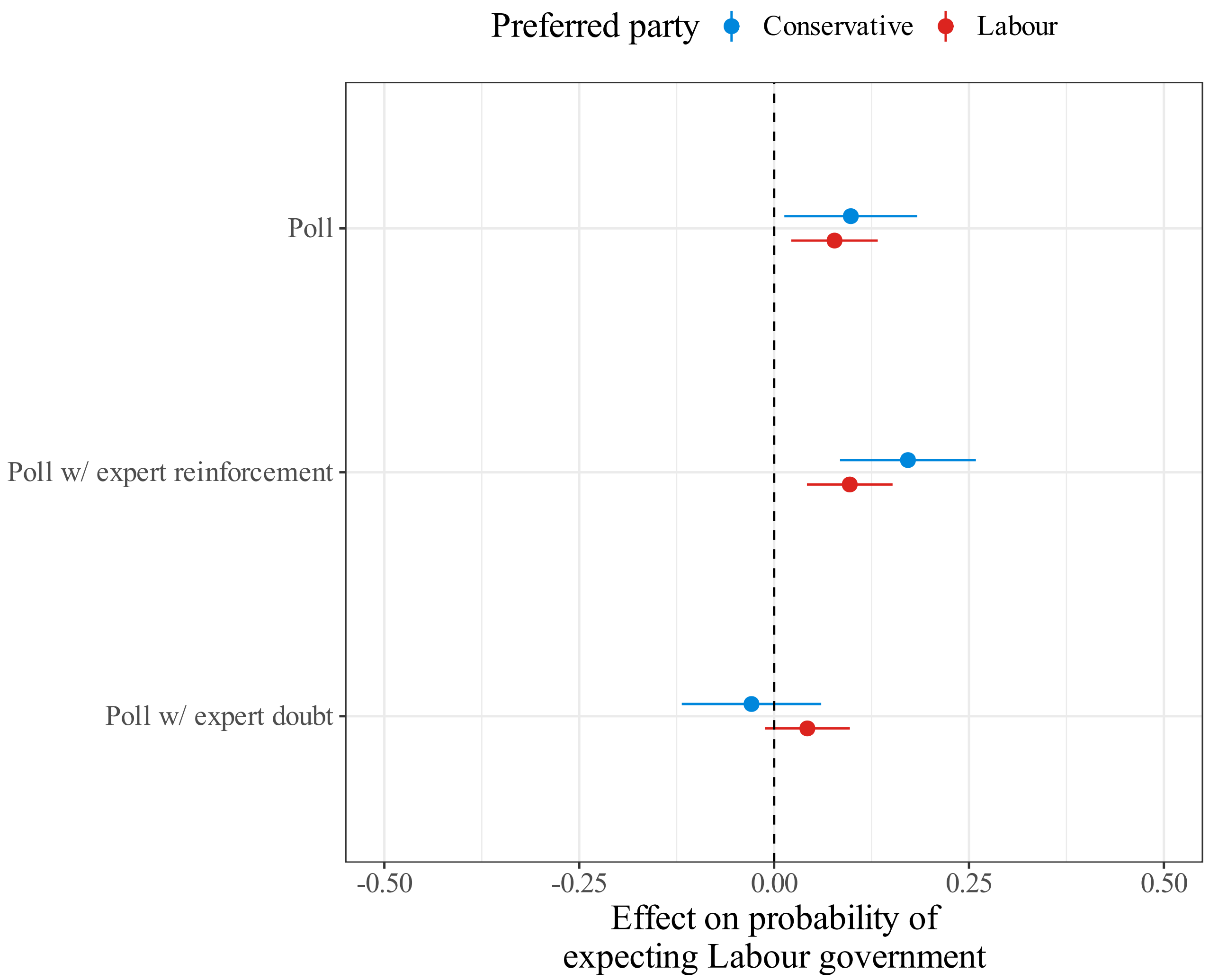

Figure 14: Effects (and 95% CI) of treatment conditions on probability of expecting Labour government, versus pure control condition.

Figure 15 displays the interaction effect between hope/optimism and treatment on the probability of choosing a Labour government over a Conservative government as most likely, in Labour and Conservative

preferrers separately. The results confirm the negative interaction effect of hope discussed in the main text, and fail to support the robustness of the positive interaction effect of optimism. All treatments have significant positive effects on Labour supporters' probability of picking a Labour government as most likely when they score *low* in hope, but those effects disappear when they are *high* in hope.

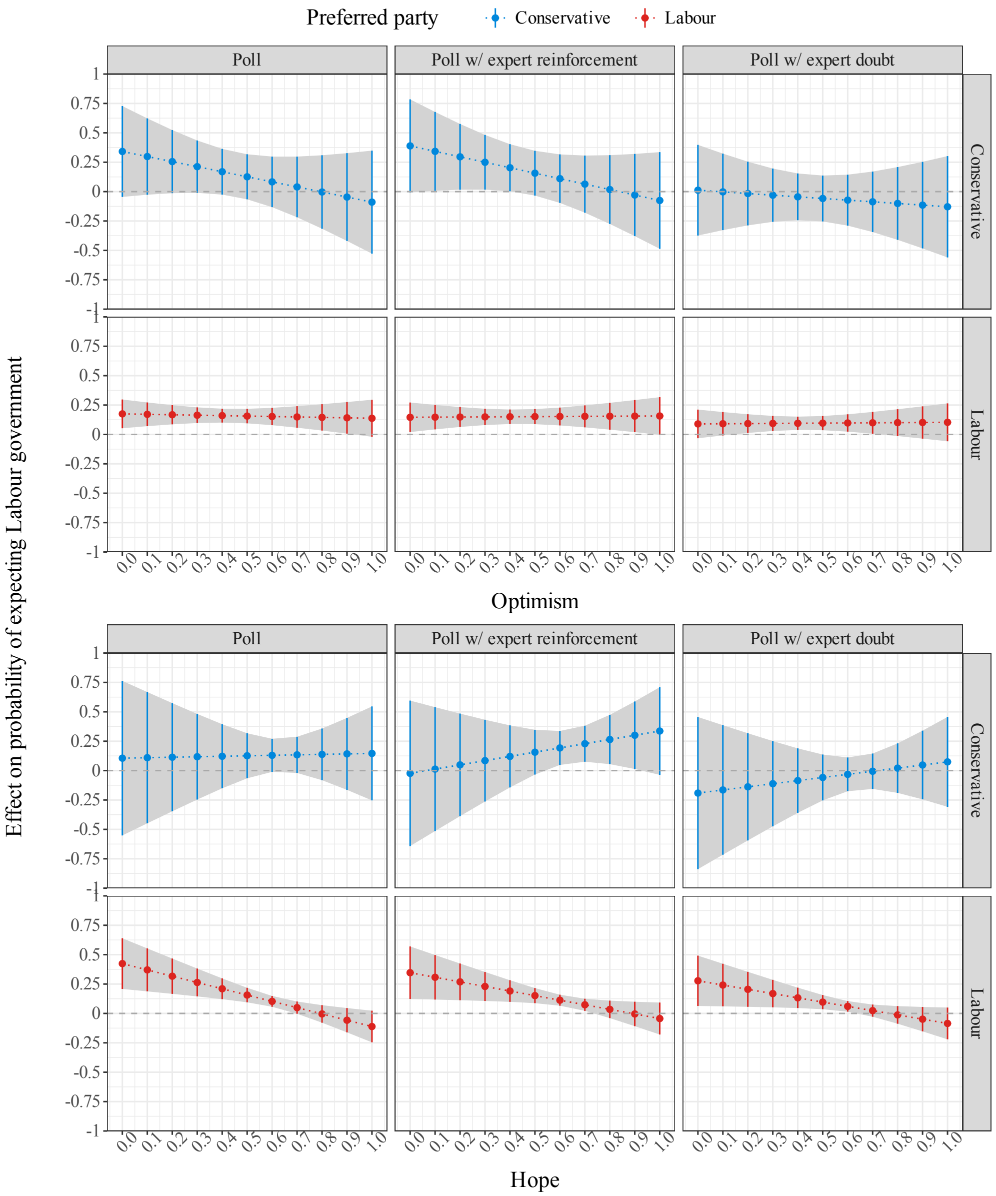

Figure 15: Effects (and 95% CI) of treatment conditions on probability of expecting Labour government, versus pure control condition, by levels of optimism/hope, controlling for age, gender, and ethnicity.

4.7 Pooled analyses

To further assess the robustness of the findings discussed in the main text, we conduct an alternative analysis in which we model the interactive effect between optimism/hope and treatment on electoral expectations, pooling together all treatments and pooling together both party supporters. The results, in Figure 16 further reinforce the robustness of the negative interaction effect of hope (β = -3.01, 95% CI: -5.61, -0.40), and fail to support the robustness of the positive interaction effect of optimism. Being exposed to any treatment, holding constant which party they support, has a significant positive effect on the expectation that their preferred party will win among those low in hope, and no discernible effect on those high in hope.

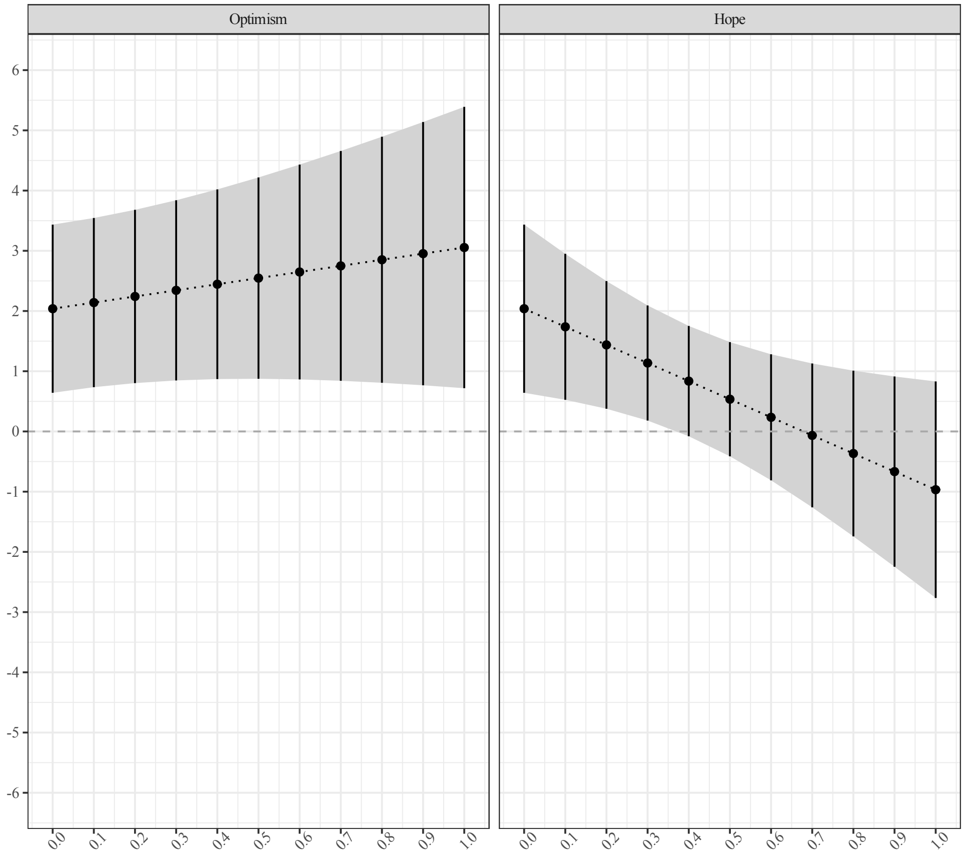

Figure 16: Effects (and 95% CI) of treatment (pooled) on perceived chances of preferred party (pooled), versus pure control condition, by levels of optimism/hope, controlling for electoral preference, age, gender, and ethnicity.

4.8 Outperform predictions items

Figure 17 displays the distributions of perceptions of whether each party would outperform experts' and polls' predictions at the election, among Conservative and Labour supporters. A majority of Conservative supporters (52.9%) expect the party to do somewhat better than predicted by polls and experts, and a large plurality of them (47.5%) expect the Labour Party to do somewhat worse than predicted. A plurality of Labour supporters (40.8%) expect their party to perform about as well as predicted (which is very well), and almost exactly equal numbers of Labour supporters expect the Conservatives to do somewhat worse (29.1%), about the same (29.9%), and somewhat better (28.6%) than predicted (which is quite badly).

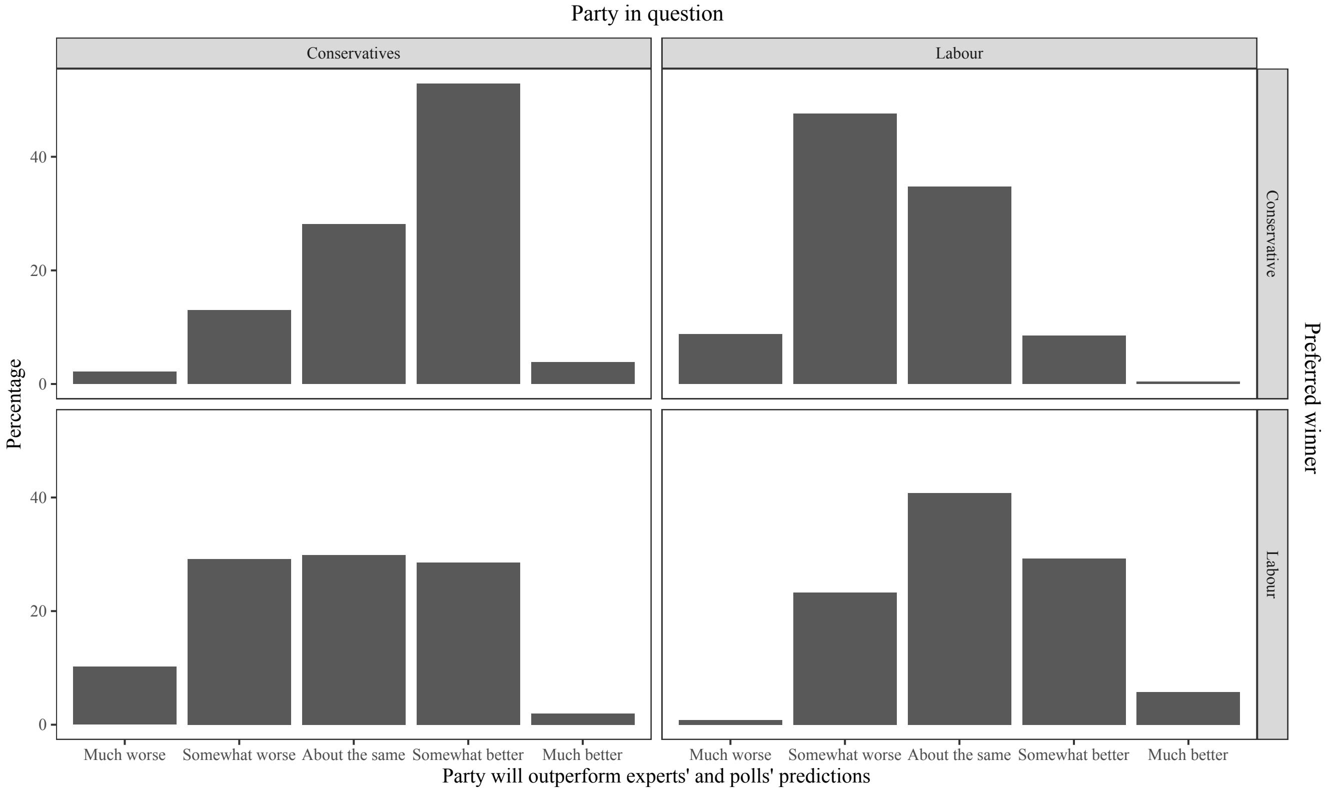

Figure 17: Distributions of responses to items asking whether parties will outperform predictions of experts and polls, by party preference.

5 Affective forecasts

5.1 Additional hypotheses

Some research has investigated people's *affective* expectations about elections: how they expect to feel after an election has taken place. There is considerable variation in such 'affective forecasts'. In contrast to the general observation that affective forecasts are exaggerated in both positive and negative directions (Wilson and Gilbert 2005), Norris, Dumville and Lacy (2011) find that, at the 2008 US presidential election, while McCain supporters overpredicted their negative affect, Obama supporters underpredicted their happiness in response to the (future) election of Barack Obama. Scheibe, Mata and Carstensen (2011) suggest these differences vary significantly across age groups. Tenenboim-Weinblatt et al. (2022) also find that exaggerated affective forecasts are more common among those with more strongly held political identities.

Optimism represents a potential further source of variation in affective forecasts. If 'the optimistic person believes that somehow – either through luck, the actions of others, or one's own actions – that his or her future will be successful and fulfilling' (Alarcon, Bowling, and Khazon 2013, 822), then optimists should be more inclined to think, whatever happens at an election, they will be OK. Optimists might also be more inclined to dwell on the potential positives, leading them especially to overpredict happiness in response to a good election outcome (Tiberius 2008; Wilson and Gilbert 2005). Simply put, optimism should moderate the relationship between partisan preferences and affective forecasts:

> **H_{6OPT}:** There is a positive interaction effect of optimism and partisan preferences on predicted future affect.

Similarly, affective forecasts may depend on levels of hope. If we assume that people have a number of future goals on which their anticipated future affect depends – that they expect to feel good if they meet their goals and bad if they do not meet them – then hopeful people will be more inclined towards positive affective forecasts, because they feel more capable of achieving those goals on which their happiness depends regardless of the election outcome. However, hope may also lead to the exaggeration of positive affective responses to good election outcomes especially, because hopeful people can see more ways in which they can make the most of positive developments to achieve their own goals. Unhopeful people, meanwhile, might underpredict happiness because they do not see ways to live a fulfilling life even under good circumstances. Simply put, hope should moderate the relationship between partisan preferences and affective forecasts:

> $\mathbf{H_{6HOPE}}$**:** There is a positive interaction effect of hope and partisan preferences on predicted future affect.

5.2 Results

Figure 18 displays the results of our analyses assessing H_{6OPT} and H_{6HOPE}. As would be expected, in every case, supporters of a party predict they will be happier if that party is in government. For example, holding all other factors constant, Labour support is associated with nearly 4 points higher (β = 3.93, 95% CI: 2.97, 4.90) predicted affect on the 0-10 scale under a Labour majority government, and with nearly 4 points lower (β = -3.88, 95% CI: -4.74, -3.02) predicted affect under a Conservative majority. Party preferences are strongly associated with predicted affect under different election outcomes.

In no case does optimism have either a main effect on predicted affect, or an interactive effect with party preferences. Across levels of optimism, predicted affect varies little, and the difference between partisans'

predicted affect barely changes. There is very little support here for H_{6OPT}.

However, in two cases, hope does matter for affective forecasts. Hope is associated with higher predicted affect under a Conservative majority (β = 2.02, 95% CI: 0.63, 3.40) and Conservative-led coalition (β = 2.18, 95% CI: 0.86, 3.49). In particular, hope in these cases is associated with higher predicted affect among Conservative supporters, but not among Labour supporters, corresponding to significant interaction effects under a Conservative majority (β = -1.72, 95% CI: -3.35, -0.08) and Conservative-led coalition (β = -2.44, 95% CI: -3.99, -0.89). These interaction effects are negative because our party preference variable is coded with Conservative as the reference category – they are the change in the effect of hope associated with being a Labour, rather than Conservative supporter. Therefore, although their sign is negative, these interaction effects are positive in the sense meant in H_{6HOPE}, so we find support for that hypothesis.

6 Preparedness

6.1 Exploratory hypotheses

We included items from the Psychological Preparedness for Disaster Threat Scale as measures of preparedness, on the basis that as a psychological disposition it might function inversely to hope and optimism. As the saying goes, 'hope for the best, prepare for the worst'. In particular, we consider the possibility that people who consider themselves better prepared for potential bad futures might in general expect more negative future outcomes. The analyses below therefore address the following, purely exploratory, hypotheses:

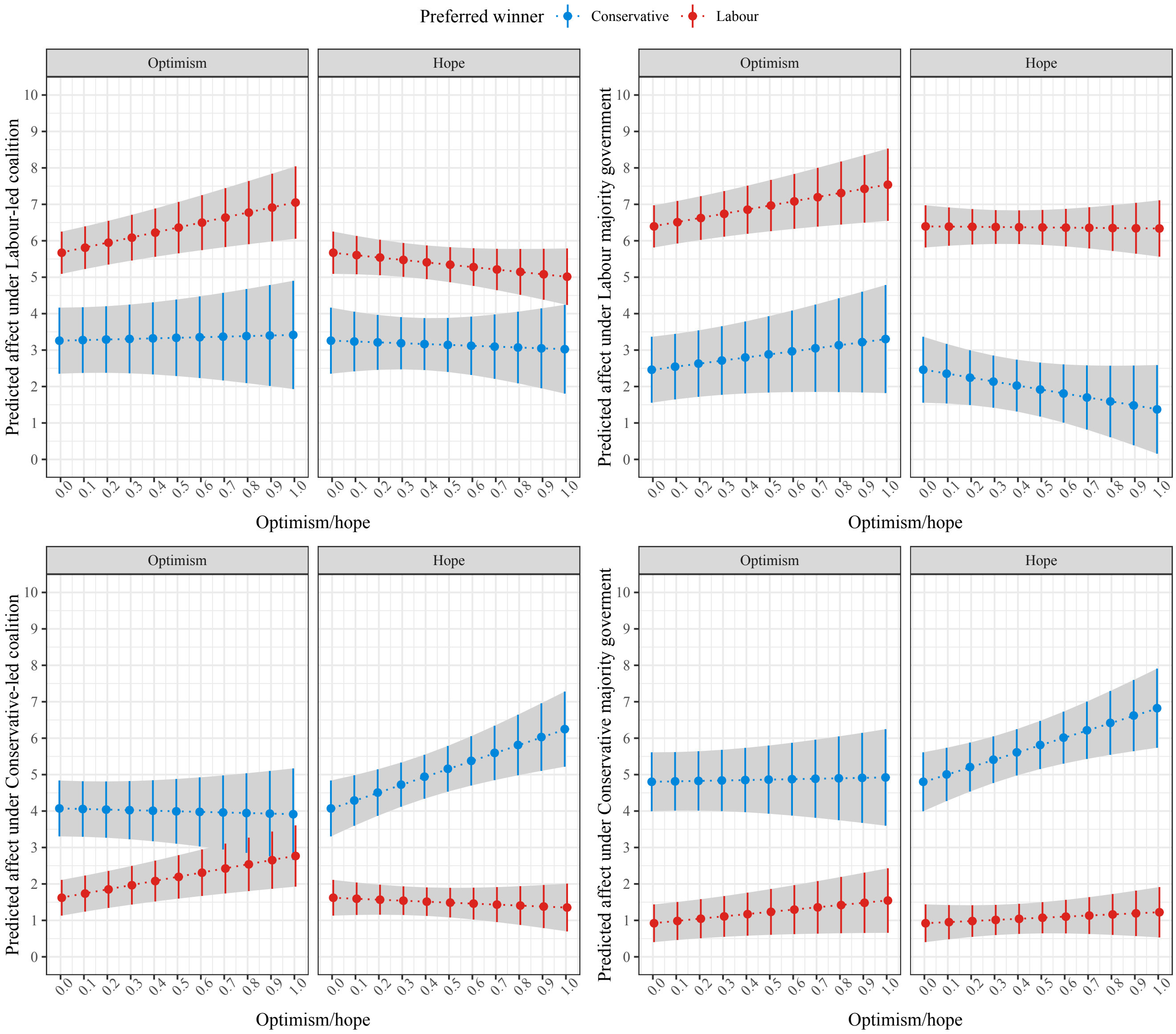

Figure 18: Predicted affect (and 95% CI) under each possible election outcome, among Labour and Conservative supporters, by levels optimism and hope, with controls for treatment condition, age, gender, and ethnicity.

H_{1PREP}: There is a negative effect of preparedness on politically relevant valence expectations.

6.2 Results

Figure 19 displays the effect of preparedness on prospective evaluations about the likelihood of positive and negative outcomes across a range of domains. Preparedness is only significantly associated with perceptions of the likelihood of getting a promotion: people who consider themselves more prepared perceive their chances of getting a big promotion at work soon significantly lower ($\beta =$ -1.41, 95% CI: -2.31, -0.50). For none of the collective, societal/political outcomes does preparedness affect evaluations. Figure 20 displays the equivalent effects on our get better/worse prospective evaluations items, and again finds that preparedness is not associated with more negative, or indeed more positive, evaluations in any case. In summary, we find no support for H_{1PREP}.

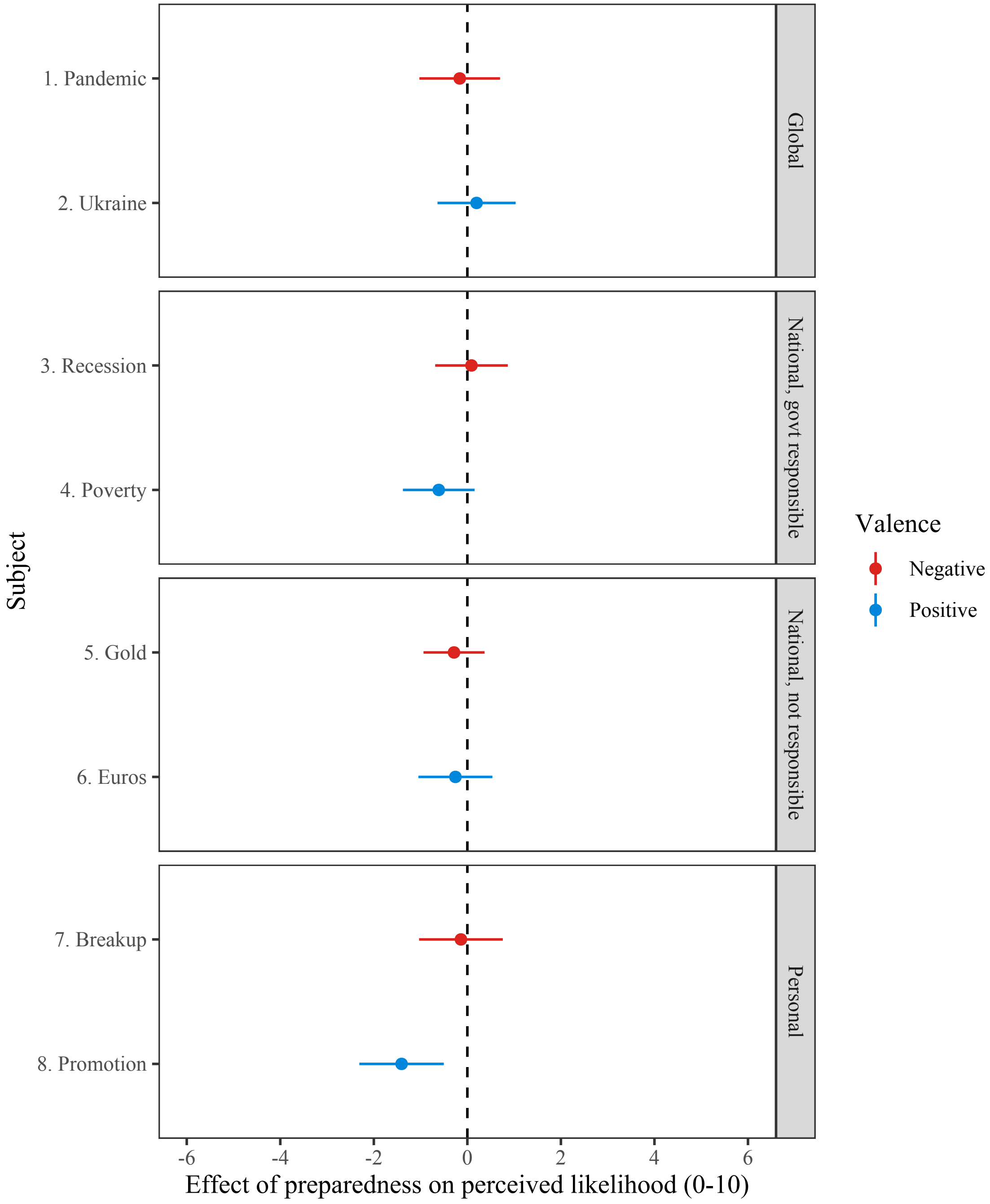

Figure 19: Effect (and 95% CI) of preparedness on perceived likelihood of future outcomes, with controls for optimism, hope, party preference, age, gender, and ethnicity.

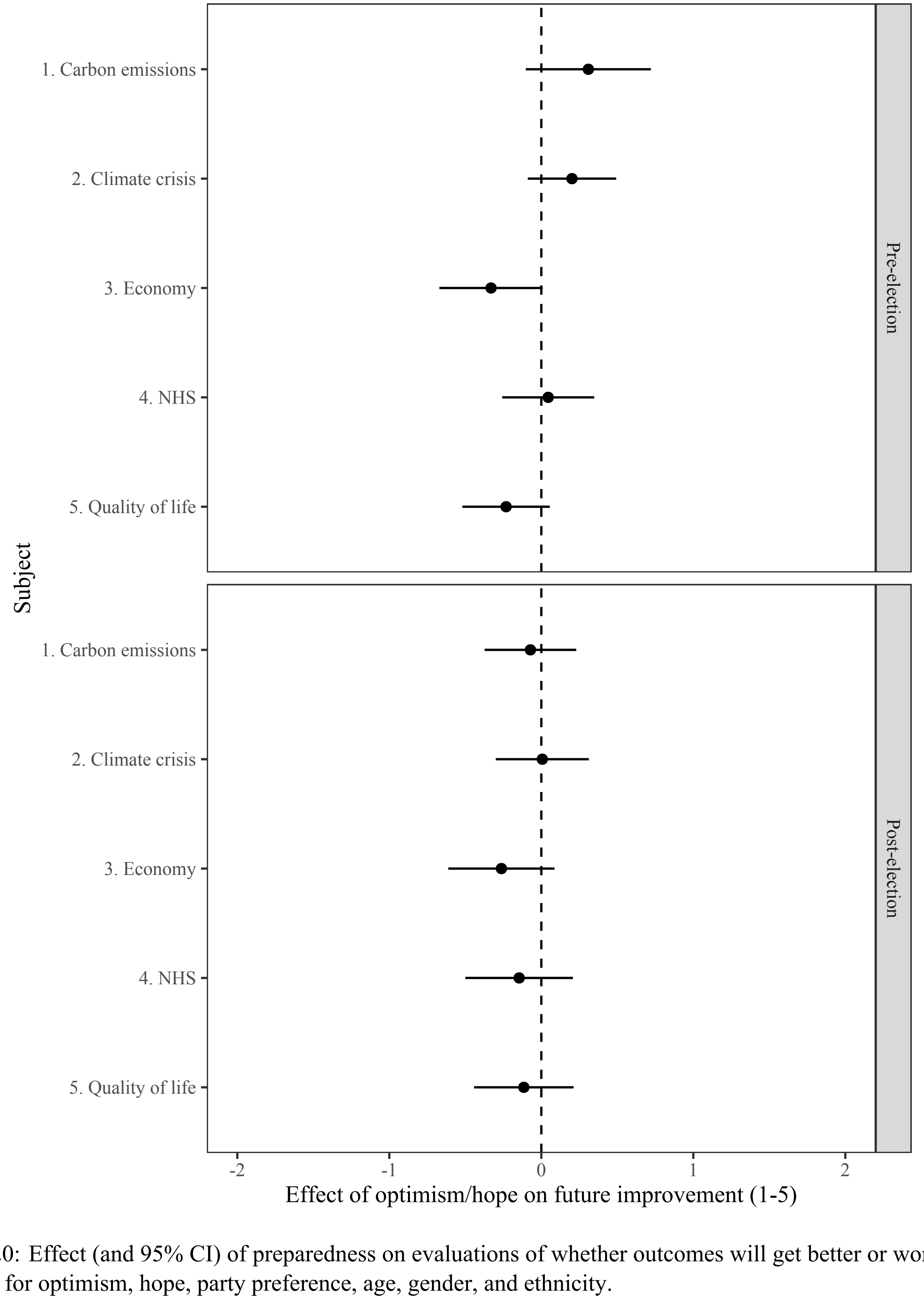

Figure 20: Effect (and 95% CI) of preparedness on evaluations of whether outcomes will get better or worse, with controls for optimism, hope, party preference, age, gender, and ethnicity.

References

Alarcon, Gene M, Nathan A Bowling, and Steven Khazon. 2013. "Great Expectations: A Meta-Analytic Examination of Optimism and Hope." *Personality and Individual Differences* 54 (7): 821–27.

Bailey, Jack. 2021. "Political Surveys Bias Self-Reported Economic Perceptions." *Public Opinion Quarterly* 85 (4): 987–1008. https://doi.org/10.1093/poq/nfab054.

Bürkner, Paul-Christian, and Matti Vuorre. 2019. "Ordinal Regression Models in Psychology: A Tutorial." *Advances in Methods and Practices in Psychological Science* 2 (1): 77–101. https://doi.org/10.1177/2515245918823199.

Liddell, Torrin M., and John K. Kruschke. 2018. "Analyzing Ordinal Data with Metric Models: What Could Possibly Go Wrong?" *Journal of Experimental Social Psychology* 79: 328–48. https://doi.org/10.1016/j.jesp.2018.08.009.

McElreath, Richard. 2020. *Statistical Rethinking: A Bayesian Course with Examples in R and Stan*. Second. London: CRC Press.

Norris, Catherine J, Amanda G Dumville, and Dean P Lacy. 2011. "Affective Forecasting Errors in the 2008 Election: Underpredicting Happiness." *Political Psychology* 32 (2): 235–49.

Scheibe, Susanne, Rui Mata, and Laura L Carstensen. 2011. "Age Differences in Affective Forecasting and Experienced Emotion Surrounding the 2008 US Presidential Election." *Cognition & Emotion* 25 (6): 1029–44.

Tenenboim-Weinblatt, Keren, Christian Baden, Tali Aharoni, and Maximilian Overbeck. 2022. "Affective Forecasting in Elections: A Socio-Communicative Perspective." *Human Communication Research*.

Tiberius, Valerie. 2008. *The Reflective Life: Living Wisely With Our Limits*. Oxford: Oxford University

Press.

Wilson, Timothy D, and Daniel T Gilbert. 2005. "Affective Forecasting: Knowing What to Want." *Current Directions in Psychological Science* 14 (3): 131–34.
